# Understanding the effects of nutrition‐sensitive agriculture interventions with participatory videos and women's group meetings on maternal and child nutrition in rural Odisha, India: A mixed‐methods process evaluation

**DOI:** 10.1111/mcn.13398

**Published:** 2022-07-19

**Authors:** Audrey Prost, Helen Harris‐Fry, Satyanarayan Mohanty, Manoj Parida, Sneha Krishnan, Emily Fivian, Suchitra Rath, Nirmala Nair, Naba K. Mishra, Shibanath Padhan, Ronali Pradhan, Satyapriya Sahu, Jolene Skordis, Heather Danton, Peggy Koniz‐Booher, Emma Beaumont, Philip James, Elizabeth Allen, Diana Elbourne, Suneetha Kadiyala

**Affiliations:** ^1^ Institute for Global Health University College London London UK; ^2^ Department of Population Health London School of Hygiene & Tropical Medicine London UK; ^3^ D‐COR Consulting Pvt. Ltd. Bhubaneshwar India; ^4^ Jindal School of Environment and Sustainability Jindal Global University Haryana India; ^5^ Ekjut Chakradharpur Jharkhand India; ^6^ Voluntary Association for Rural Reconstruction and Appropriate Technology (VARRAT) Kendrapara India; ^7^ Digital Green New Delhi India; ^8^ JSI Research & Training Institute, Inc. Arlington Virginia USA; ^9^ Department of Medical Statistics London School of Hygiene & Tropical Medicine London UK

**Keywords:** child nutrition, cluster randomised controlled trial, diet, maternal nutrition, qualitative methods, socioeconomic factors

## Abstract

A trial of three nutrition‐sensitive agriculture interventions with participatory videos and women's group meetings in rural Odisha, India, found improvements in maternal and child dietary diversity, limited effects on agricultural production, and no effects on women and children's nutritional status. Our process evaluation explored fidelity, reach, and mechanisms behind interventions' effects. We also examined how context affected implementation, mechanisms, and outcomes. We used data from intervention monitoring systems, review notes, trial surveys, 32 case studies with families (*n* = 91 family members), and 20 group discussions with women's group members and intervention workers (*n* = 181 and 32, respectively). We found that interventions were implemented with high fidelity. Groups reached around half of the mothers of children under 2 years. Videos and meetings increased women's knowledge, motivation and confidence to suggest or make changes to their diets and agricultural production. Families responded in diverse ways. Many adopted or improved rainfed homestead garden cultivation for consumption, which could explain gains in maternal and child dietary diversity seen in the impact evaluation. Cultivation for income was less common. This was often due to small landholdings, poor access to irrigation and decision‐making dominated by men. Interventions helped change norms about heavy work during pregnancy, but young women with little family support still did considerable work. Women's ability to shape cultivation, income and workload decisions was strongly influenced by support from male relatives. Future nutrition‐sensitive agriculture interventions could include additional flexibility to address families’ land, water, labour and time constraints, as well as actively engage with spouses and in‐laws.

## Key messages

- In rural eastern India, participatory videos and women's group meetings on agriculture and maternal and child nutrition increased women's knowledge, motivation and confidence to improve their and their children's diets.
- Given strong constraints linked to small landholdings, poor access to water and gender norms which meant that in‐laws' and husbands' assent or support were often required for cultivation decisions, many women responded to interventions by increasing rainfed homestead garden cultivation for consumption rather than cultivating for income.
- Women's and children's diets may have improved because of discussions about nutrition and an increase in homestead garden cultivation. These dietary changes alone were likely insufficient to improve women's and children's nutritional status.
- The interventions’ ability to influence the adoption of nutrition‐sensitive agriculture practices could be improved by being family‐centric: understanding women's decision‐making power in a family context, using tailored problem‐solving to address households' individual constraints to cultivation, and including women's husbands and in‐laws.

## Graphical abstract

In rural eastern India, participatory videos and women's group meetings on agriculture and maternal and child nutrition increased women's knowledge, motivation and confidence to improve their and their children's diets. Despite the intervention's emphasis on addressing social and structural constraints to cultivation, small landholdings, poor access to water and the need for in‐laws' or husbands' support to make changes to cultivation patterns led many women to respond to interventions by increasing rainfed homestead garden cultivation for consumption rather than cultivating for income. The interventions' ability to influence the adoption of nutrition‐sensitive agriculture practices could be improved by being family‐centric: understanding women's decision‐making power in a family context, using tailored problem‐solving to address households' individual constraints to cultivation, and including women's spouses and in‐laws.

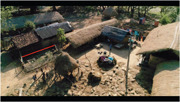

## 1 INTRODUCTION

Achieving the second sustainable development goal (zero hunger) requires making agriculture ‘work’ for nutrition (Food and Agriculture Organization of the United Nations, 2018; Ruel et al., 2013). An ideal gender‐ and nutrition‐sensitive agriculture intervention would increase agricultural productivity, boost women's agency, and promote nutritious crops that can be consumed by women or sold for income without increasing workload or infection risk (Kadiyala et al., 2014). Existing nutrition‐sensitive agriculture interventions have had mixed effects on agriculture production, income, women's empowerment, workload and nutritional status (Girard et al., 2012; Johnston et al., 2018; Ruel et al., 2018), suggesting the need for further research (Sharma et al., 2020).

Upscaling Participatory Action and Videos for Agriculture and Nutrition (UPAVAN) was a four‐arm cluster‐randomised controlled trial testing three nutrition‐sensitive agriculture interventions with participatory videos and women's group meetings to improve maternal and child nutrition in rural villages of Odisha, India. The interventions aimed to improve two primary outcomes: dietary diversity among children aged 6–23 months and the body mass index (BMI) of mothers or female primary caregivers of children aged 0–23 months. Secondary outcomes were maternal dietary diversity and weight‐for‐height z‐score of children aged 0–23 months. UPAVAN interventions sought to influence these outcomes by supporting seasonally appropriate, locally feasible production and diversity of nutritious or income‐generating foods, improving women's decision‐making power in agriculture activities and reducing women's workload in agriculture.

In this study, we report the trial's process evaluation, which aimed to understand the fidelity of the interventions' implementation, their reach, underlying mechanisms and contextual factors affecting implementation, mechanisms and outcomes (Moore et al., 2015).

## 2 METHODS

### 2.1 Setting

UPAVAN took place in 148 clusters of four rural blocks of Keonjhar district, Odisha: Patna, Ghatgaon, Harichandanpur and Keonjhar‐Sadar. In the trial's baseline survey (November 2016 to January 2017), 45% of women had a BMI < 18.5 kg/m^2^ (*N* = 4480) and 15% of children aged 0–23 months were too thin for their length (weight‐for‐length < −2 SD) (*N *= 4430) (Kadiyala et al., 2021).

The majority (81%) of households owned less than 1 hectare of land. Fifty‐eight percent were from indigenous communities (scheduled tribes), 30% from other backward class communities and 9% from scheduled castes (Kadiyala et al., 2021). Indigenous families often have lower incomes, smaller landholdings, and poorer health compared to other social groups (Debasree, 2015; Kumar et al., 2011; Malik, 2020; Rath, 2005; Government of India Ministry of Health and Family Welfare & Ministry of Tribal Affairs, 2018).

**Figure 1:**
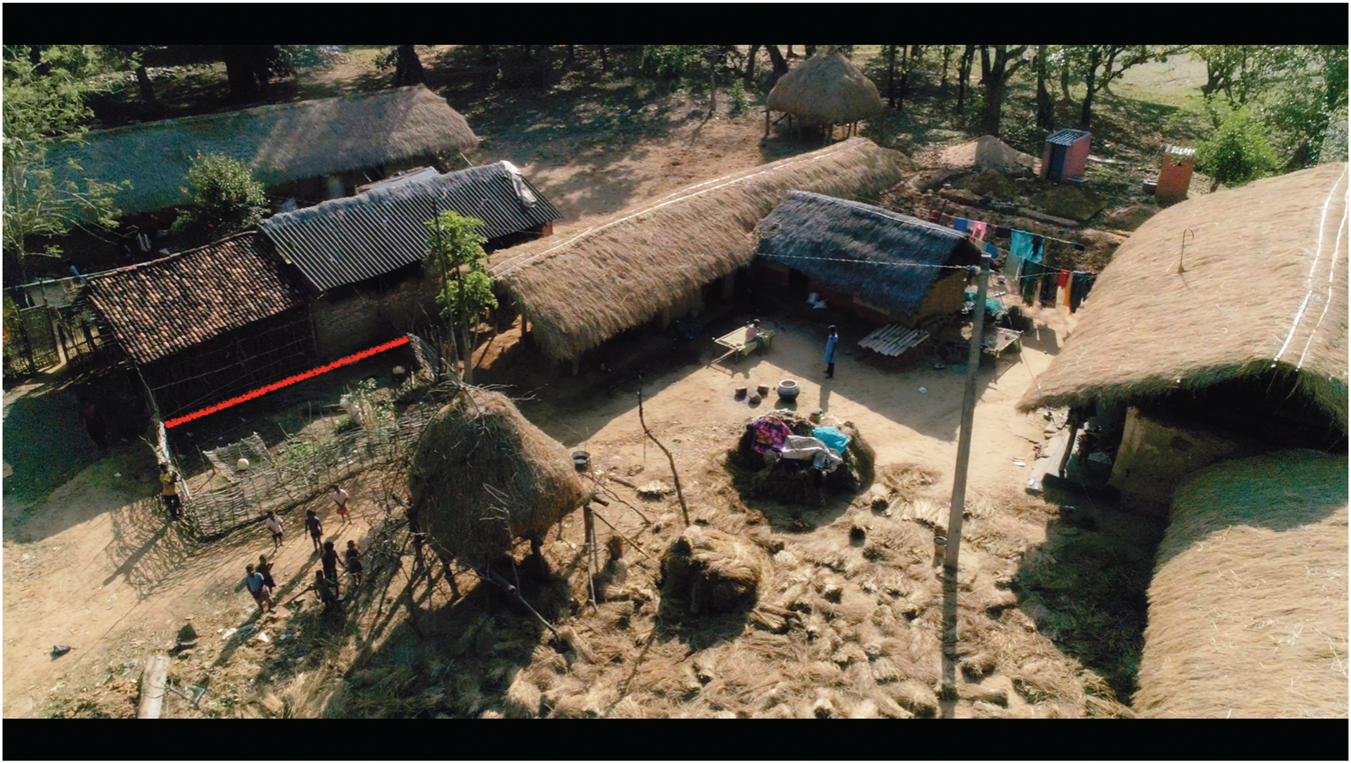
Homestead garden in Keonjhar, Odisha

Families in the study areas had a mean of 5.2 members (SD: 1.7). Most practised subsistence agriculture alongside wage labour. Around half (48%) of crops came from rainfed *kharif* (monsoon) agriculture and 70% of vegetable cultivation required irrigation (District Level Implementation Committee Pradhan Mantri Krishi Sinchayee Yojana, 2016). Between 60% and 80% of families had homestead gardens of 5–9 m^2^, mostly tended by women (Figure 1). The trial baseline suggested that the median annual paddy yield per household (700 kg) was insufficient to feed most families. Eighty per cent of households in all arms produced paddy, 52% of households caught fish, 46% reared chickens, 9% produced dairy, and less than 16% produced vegetables. The baseline also found that 67% of women were involved in some or all decisions about crops grown for household consumption and livestock, but only 18% took part in decisions about crops grown for income generation. Newly married women worked an average of 11 h per day in and on the farm. Mothers‐in‐law had a strong influence over their diets and workloads.

### 2.2 Interventions

The UPAVAN trial tested three participatory nutrition‐sensitive agriculture interventions to improve maternal and child nutrition. The interventions included fortnightly women's group meetings followed by home visits over 32 months, as described in Panel 1 and in the intervention video (Sharma, 2021). All interventions were offered through women's self‐help groups of 15–20 members, but all women living in intervention clusters were invited to participate in UPAVAN meetings, whether they were self‐help group members or not. UPAVAN meetings therefore included a mix of self‐help group members who contributed to the group fund, and nonself‐help group members who did not contribute financially. Although usual self‐help group activities (credit, loans and savings) were generally kept separate from the UPAVAN meetings, on two occasions (at the start and 6 months later) voluntary association for rural reconstruction and appropriate technology (VARRAT) led self‐help group‐strengthening activities that aimed to increase membership and record‐keeping skills.

Panel 1 All UPAVAN interventions used a participatory video approach designed by Digital Green, an international nongovernmental organisation. Community members identified locally relevant agriculture‐related topics and developed packages of practices—key actions to improve agricultural practices—to discuss in videos. Community resource persons who were local community members were trained in storyboarding, video production and editing. Other community members were then filmed demonstrating and discussing the key practices. Salaried Community Service Providers showed these videos to women's groups, pausing videos at specified timepoints to stimulate discussion of the practices. Groups were then encouraged to discuss the feasibility, interest, apprehension or experience and possible barriers in adopting the practices. Facilitators visited group members in their homes or farms and asked whether they adopted the practices. Community Service Providers monitored video viewership, knowledge recall and adoption of practices. These data were collated in a monitoring system, and qualitative feedback was gathered during review meetings. This information was used by local implementers to identify future video content.

We adapted this participatory video approach to make it nutrition‐sensitive and enhance participation, as described in depth elsewhere (Kadiyala et al., 2018). Interventions delivered in the three UPAVAN arms built on the participatory video approach and were as follows:

AGRI arm: fortnightly women's groups viewing and discussing videos to promote nutrition‐sensitive agricultural practices developed through participatory processes in the local area. Nutrition‐sensitive agriculture videos promoted practices aimed at increasing the availability of nutritious foods, increasing household income, improving women's decision‐making in agriculture and reducing pregnant and breastfeeding women's workloads. Group participants who were pregnant or had a child (<2 years) were meant to receive follow‐up visits at home after each video to check whether they retained the messages, discuss if mothers or household members had adopted practices shown in the videos and encourage continued participation in the intervention.

AGRI‐NUT: as in AGRI, but with the addition of nutrition‐specific videos on maternal and child nutrition focused on age‐appropriate child feeding practices, care during child illness, and maternal diets and rest.

AGRI‐NUT‐PLA: fortnightly women's groups viewing and discussing participatory nutrition‐sensitive agriculture and nutrition‐specific videos combined with a cycle of Participatory Learning and Action (PLA meetings, with follow‐up home visits. The PLA meeting cycle with women's groups had four phases. In phase 1, group members identified and prioritised nutrition problems. In phase 2, they explored the causes and effects of prioritised problems, planned locally feasible strategies to address these, decided on roles and responsibilities for implementing the strategies, and shared their learning with the wider community. In phase 3, groups implemented their strategies. Finally, in phase 4, they evaluated the process.

In the first of the UPAVAN intervention arms (AGRI), local salaried facilitators called Community Service Providers disseminated videos on nutrition‐sensitive agriculture and facilitated related discussions with women's groups. Community Resource Persons trained as videographers filmed farmers and government frontline workers discussing or demonstrating core practices. Videos aimed to promote uptake or improvements in the cultivation of seasonally appropriate crops and livestock (e.g., introducing carrot cultivation, improving spacing between spinach plants, sowing seeds for cowpea cultivation, vaccinating goats, rearing chickens), develop skills (e.g., household budgeting, crop planning) and encourage group‐based activities (e.g., buying seeds in bulk).

Video content was selected using social and agronomic information gathered during formative research, and by responding to requests or barriers faced by group participants, including land or water scarcity (Aakesson et al., 2017; Harris‐Fry et al., 2020). For example, some videos focused on cultivating Indian spinach with wastewater. Others described how to grow mushrooms, which require minimal labour and are of high economic value.

In the second intervention arm (AGRI‐NUT), women's groups viewed and discussed nutrition‐sensitive agriculture videos and videos promoting nutrition‐specific maternal, infant and young child nutrition (AGRI‐NUT) designed using formative research, again under the guidance of Community Service Providers. Videos also described ways to overcome social and economic barriers to adopting these changes. For example, one video described a mother‐in‐law becoming an advocate for the consumption of Indian spinach, challenging widely‐held beliefs about spinach being harmful for children and pregnant women. Other videos gave recipes and demonstrations on the appropriate diversity, quantity, frequency and consistency of complementary foods for young children.

In the third intervention arm (AGRI‐NUT‐PLA), women's groups viewed and discussed nutrition‐sensitive agriculture videos, but also took part in a Participatory Learning and Action cycle of meetings in which they identified, prioritised and addressed problems related to maternal and child nutrition in four phases (Kadiyala et al., 2018). In phase one, groups identified and prioritised problems related to maternal and child nutrition and hygiene. In phase 2, they sought to understand the causes of prioritised problems and identify locally appropriate strategies to address them. In phase 3, groups implemented and reviewed chosen strategies. In the final phase, groups evaluated activities and discussed strategies which they thought were impactful or difficult. Participatory Learning and Action meetings were either discussions without videos using techniques relevant to each phase of the cycle, or participatory videos on nutrition‐specific topics that were developed as part of the Participatory Learning and Action process. The nutrition‐specific videos used in this arm were therefore different to those in the AGRI‐NUT arm. In the AGRI‐NUT‐PLA arm, groups had on average one nutrition‐sensitive agriculture video and one Participatory Learning and Action meeting per month.

While creating all intervention videos, we used the transtheoretical model of behaviour change's stages of change to reinforce uptake and maintenance of core practices (Prochaska & DiClemente, 1982). Specifically, we mapped whether a proposed behaviour was new to a community (‘precontemplation’); being passively considered (‘contemplation’); actively considered (‘preparation’); first adopted (‘action’); continued (‘maintenance’) or modelled for others in the community (‘termination’) (Harris‐Fry et al., 2020; Prochaska & DiClemente, 1982).

We hypothesised that one or more of the three UPAVAN interventions would improve nutrition‐sensitive agriculture and nutrition‐specific practices through six pathways:

(1) mothers and children would consume nutrient‐rich foods produced;
(2) households would invest income from agriculture to improve nutrition;
(3) pregnant and breastfeeding women would reduce their energy expenditure and hours worked;
(4) women would influence and make more decisions about agricultural activities, workload and use of income;
(5) pregnant and breastfeeding women would have adequate diets;
(6) children would have adequate diets.

**Figure 2:**
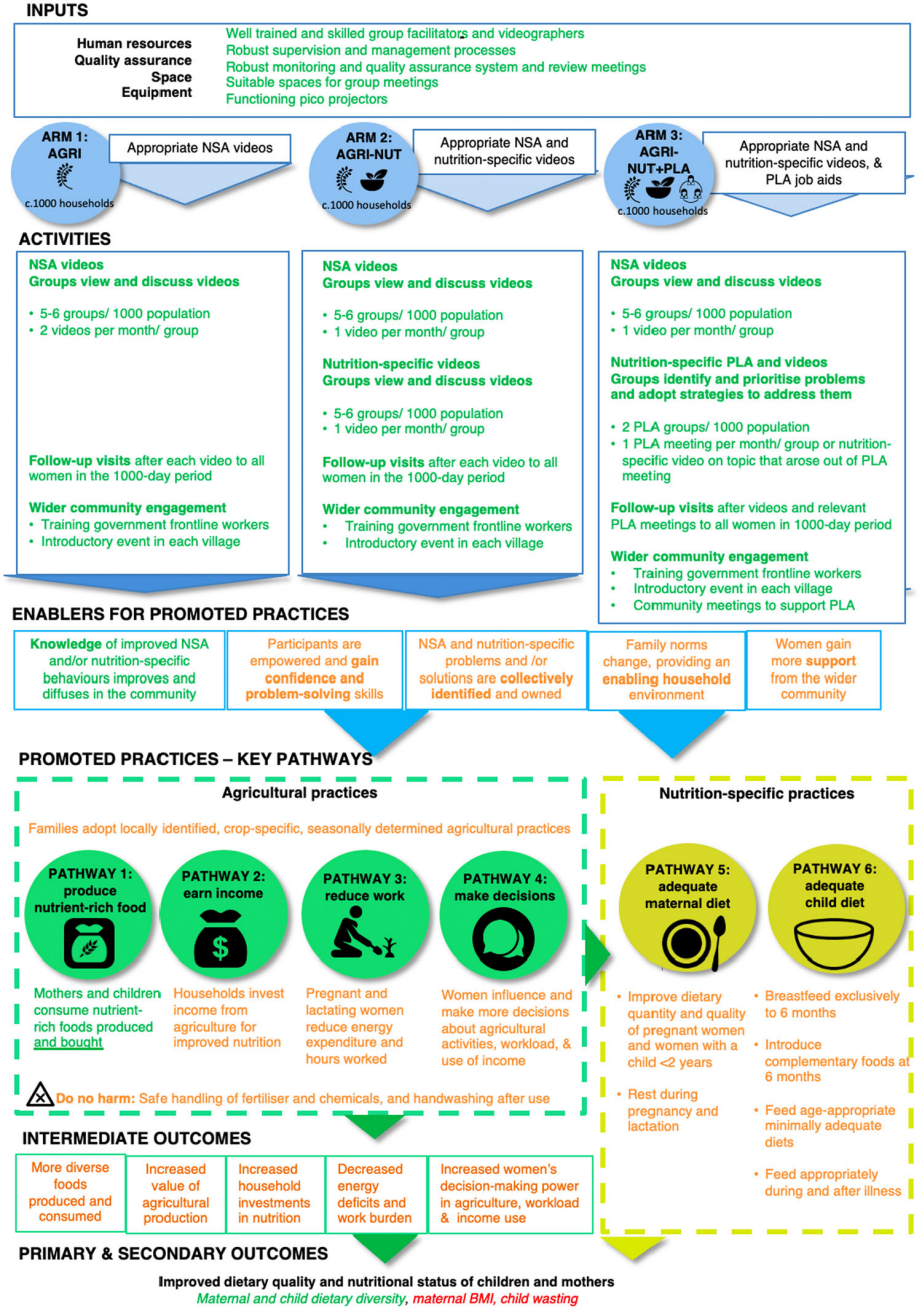
Theory of change, with the strength of evidence for the activation of components.

These changes would in turn lead to improved nutritional status for women and children. Our theory of change (Figure 2) described these pathways and the intervention inputs, activities and enablers, with broad arrows reflecting hypothesised causal mechanisms. We used the term ‘mechanism’ to refer to both enablers and pathways in Figure 2. We anticipated that improvements in nutrition outcomes would occur through changes to multiple rather than single enablers and practices. For example, women might require both knowledge about nutrition‐sensitive agricultural practices and an enabling household environment to adopt new practices. In addition, changes to multiple practices would be required to affect an outcome such as BMI. We also anticipated that change would not be linear. For example, some practice‐outcome relationships could be mutually reinforcing: a woman gaining income from selling mushrooms after watching a related video might become more motivated to attend additional video dissemination and adopt other practices.

### 2.3 Trial results

Detailed UPAVAN trial results have been reported elsewhere (Kadiyala et al., 2021). Briefly, the trial was powered to detect a 9% absolute difference in the proportion of children achieving minimum dietary diversity (four or more food groups) and a 0.3 kg/m^2^ increase in maternal BMI in each intervention arm versus a control arm. It found an increase in the proportion of children achieving minimum dietary diversity in both arms where groups discussed maternal and child nutrition (AGRI vs. control: 1.06 [0.91, 1.23]; AGRI‐NUT vs. control: 1.19 [1.03, 1·37]; AGRI‐NUT‐PLA vs. control: 1.27 [1.11, 1.46]), but no effect on maternal BMI (AGRI: −0.05 [−0.34, 0.24]; AGRI‐NUT: 0.04 [−0.26, 0.33]; AGRI‐NUT‐PLA: −0.03 [−0.30, 0.23]). There were significant or borderline effects on the secondary outcome of maternal minimum dietary diversity in all three intervention arms (AGRI vs. control: 1.21 [1.01, 1.45]; AGRI‐NUT vs. control: 1.16 [0.98, 1.38]; AGRI‐NUT‐PLA vs. control: 1.30 [1.10, 1.53]), but no effect on child wasting in any arm (AGRI: 0.95 [0.73, 1.24]; AGRI‐NUT: 0.96 [0.72, 1.29]; AGRI‐NUT‐PLA: 0.96 [0.73, 1.26]). The AGRI intervention increased women's decision‐making power as well as the total and net annual value of agricultural production compared to the control arm. The trial found no consistent effects on agricultural production diversity, gender parity in decision‐making for agriculture and health, household expenditure or women's work in any arm.

### 2.4 Process evaluation objectives

Our mixed‐methods process evaluation had three objectives:

(1) To assess the fidelity of the UPAVAN interventions' implementation and their reach
(2) To clarify the mechanisms behind the interventions' effects
(3) To identify contextual factors associated with variations in implementation, mechanisms and outcomes

### 2.5 Data collection

We used six sources of quantitative and qualitative data, as described in Table 1. Quantitative data included the trial surveys and information from routine monitoring. Trial surveys captured data on group attendance and home visits 6 months before the survey among mothers of children under 2, as well as household agricultural production diversity, gender parity in decision‐making for agriculture and health, household expenditure, women's work, maternal and child dietary diversity, maternal BMI and child wasting. Methods for these surveys are described fully in the trial article (Kadiyala et al., 2021).

**Table 1:** Data sources

*N*	Data source
1	Routine monitoring data collected by VARRAT on the number of video disseminations, PLA meetings and home visits held during the 32‐month intervention period (2017–2019)
2	UPAVAN trial surveys, which included 4480 mothers of children under 2 years and 4473 spouses at baseline, and 4291 mothers and 4287 spouses at endline (Kadiyala et al., 2021)
3	Thirty‐two family case studies including semistructured interviews of pregnant women, mothers of children under two, their husbands and in‐laws (8 in the AGRI arm, 12 in the AGRI‐NUT arm, and 12 in the AGRI‐NUT‐PLA arm or a total of 91 interviews).
4	Seventeen focus group discussions with a total of 181 self‐help group members (5–6 groups per arm).
5	Three focus group discussions (one per intervention arm) with a total of 32 VARRAT staff. These included Community Service Providers, VARRAT's frontline workers, and their supervisors.
6	Five annual reports and six reports of intervention team review meetings.

Abbreviation: VARRAT, voluntary association for rural reconstruction and appropriate technology.

Supporting Information: Figure 1 is a timeline for the interventions and data collection. Five D‐COR team members fluent in Odia collected the qualitative data over two phases (March to April 2018 and March to April 2019) with support from VARRAT, Ekjut and the London School of Hygiene and Tropical Medicine. We aimed for the qualitative sample to include community members who could be influenced by the intervention (self‐help group members, and members who were pregnant or had a child aged under 2 years, their husbands and in‐laws), as well as intervention workers. We also aimed for representation from communities across the trial's three intervention arms. We therefore, selected five trial clusters per intervention arm, broadly following the trial's stratification: one with a low proportion of scheduled caste and/or indigenous families (<30%); two with a medium (30%–70%) proportion (one close, i.e., <10 km, and one further away, i.e., ≥10 km from the nearest town) and two with a high (>70%) proportion (one close and one further away from the nearest town). In each village, D‐COR asked for permission to attend a video dissemination meeting, and then approached pregnant women and mothers of children under 2 years to take part in semistructured interviews. These women, along with their mother‐in‐law and husband when available, were interviewed to create a case study. The case studies focused on changes to the diets of mothers (including during pregnancy) and young children and the adoption of nutrition‐sensitive agriculture practices. We sought to understand what enabled these changes, and the reasons behind nonadoption. Topic guides were developed to capture elements in the theory of change and are included in the supplementary file. Supporting Information: Table 1 shows the case study participants' characteristics.

We invited all self‐help group members from the five selected villages for focus group discussions. These discussions focused on understanding who participated in self‐help group meetings and members' experiences of the UPAVAN interventions. Group discussions conducted with Community Service Providers and supervisors explored barriers and enablers to delivering the interventions as well as community responses to interventions. We aimed for the final qualitative sample size to be around 20 group discussions with 10–15 participants each, and 30 case studies with 3–4 participants each. We felt that this would enable us to explore a variety of family circumstances and responses to interventions.

### 2.6 Analysis

D‐COR carried out near‐verbatim transcription of audio recordings and translated transcripts into English. S. M., M. P., S. K. and A. P. used a thematic approach to capture themes related to each component of the theory of change and emergent themes in Nvivo (Denzin & Lincoln, 2017). We first coded data using a framework derived directly from elements of the theory of change and captured each element under inputs, activities, enablers, promoted practices/key pathways and outcomes. We then added new nodes for emergent themes (e.g., lack of water as a barrier to taking up nutrition‐sensitive agriculture). A. P. coded case studies using the theory of change framework in Nvivo, then summarised them narratively with inputs from S. M. We discussed results with the entire UPAVAN study team to check our interpretations.

The final analysis of process evaluation data was conducted after the trial results were known. We explored fidelity in relation to the ‘inputs’ described in the theory of change, using intervention monitoring and qualitative data as well as review meeting notes. We then described the interventions' reach and contextual factors that might affect it using the trial survey's coverage data and qualitative data. Finally, we reviewed mechanisms related to both enablers and promoted practices along the key pathways described in Supporting Information: Table 3, using qualitative data. We analysed qualitative and quantitative data separately but triangulated these while examining all components of the intervention's theory of change. If quantitative surveys provided evidence for the activation of a component in one or more intervention arms compared to the control arm, and if the qualitative data supported this, we interpreted this as strong evidence that the component had been activated. If only one type of data supported activation, or if they suggested heterogeneous effects, we interpreted this as moderate evidence of activation and explored reasons for heterogeneity in the qualitative data. If neither the qualitative nor quantitative data supported activation, or if the component could only be measured using quantitative data and these did not suggest an effect, we interpreted this as ‘no evidence of activation’. We provide supporting data related to the activation of some components in the Supporting Information: Appendix (e.g., SD1, is ‘Supporting Data 1’ in the Supporting Information: Appendix, Table 3).

## 3 RESULTS

Figure 2 is a colour‐coded version of the intervention's theory of change, indicating components for which we found strong, moderate or no evidence of activation. We coded several components as having moderate evidence of activation because qualitative data showed that women had differing experiences, both between and within arms. We now review individual components of the theory of change. We highlight contextual factors that could explain variations in implementation, mechanisms and outcomes throughout each section. All supporting data are presented in the text or in indexed numerically in the Supporting Information: File's Table 3 (e.g., SD1 is the first row in the Supporting Information: Data Table).

### 3.1 Fidelity

UPAVAN interventions were implemented with high fidelity. VARRAT held intervention launch events with community members, local magistrates, self‐help group representatives, Panchayati Raj members, school teachers and government community‐based frontline workers (Anganwadi workers and Accredited Social Health Activists). VARRAT also organised 2‐day trainings with these frontline workers in all study areas, including the control arm. This aimed to increase collaboration between frontline workers and Community Service Providers, and ensure they had similar knowledge. Monitoring data suggested that 38.3% (646/1687) of video disseminations in the AGRI arm were attended by frontline workers, compared with 56.9% (1007/1770) in the AGRI‐NUT arm and 40.2% (533/1326) in the AGRI‐NUT‐PLA arm.

Training and supervision increased Community Service Providers' knowledge of nutrition‐sensitive agriculture, maternal and child nutrition, and their confidence in discussing these with community members (Kadiyala et al., 2021) (SD1). Community Service Providers were motivated by helping spread ‘knowledge and development’ and gaining recognition from their work in the community (SD2). Few had difficulties finding venues for video dissemination or Participatory Learning and Action meetings (SD3).

Community Service Providers successfully achieved the planned intervention coverage of groups: there was one video dissemination ‘point’ for two self‐help groups or five to six dissemination points per 1000 population (people) in the AGRI and AGRI‐NUT arms, and two Participatory Learning and Action groups per 1000 population in the AGRI‐NUT‐PLA arm. Monitoring data confirmed that the planned number of video dissemination and Participatory Learning and Action meetings were held over the intervention period. Community Service Providers also improved the inclusion of mothers‐in‐law, and pregnant and breastfeeding women in self‐help groups (SD4).

### 3.2 Reach

#### 3.2.1 Group meetings

UPAVAN aimed to reach pregnant women and mothers of children under 2 by strengthening self‐help groups. There was some evidence that this worked. In the trial's baseline data, only 19% of mothers of children under 2 years were ‘active’ in self‐help groups, which was defined as participating in meetings and discussions for the last three agricultural seasons (1 year). By contrast, at endline, around half of mothers of children under 2 years were active, including in the control arm. Between 50% and 57% of mothers had seen any video (AGRI and AGRI‐NUT) or been to any Participatory Learning and Action meeting (55% in AGRI‐NUT‐PLA) in the 6 months before the endline survey (Supporting Information: Table 2). Mean (range 0–11) number of events (videos of PLA meetings) mothers attended in the last 6 months before the endline survey were 3.3 (AGRI); 4.4 (AGRI‐NUT) and 4.6 (AGRI‐NUT +PLA).

In group discussions, self‐help group members explained that some women did not join groups because they did not have money to contribute, or conversely because they already had money and therefore had no interest in joining a self‐help group (SD5):

I [Interviewer]: Ok, why haven't they joined the group? P3 [Participant 3]: […] Maybe they didn't have money to give. Maybe they aren't able to join. […] P1: They are from well‐to‐do families but we have our needs, we want to do something by joining in this group. Because they have sufficient money to manage, they do not want to go out. (Self‐help group discussion 1, AGRI)

It is possible that some women did not realise they were not required to contribute funds to the self‐help group, and that this could have acted as a deterrent to poorer women. Community Service Providers and group members described several other influences on meeting participation. Efforts to invite women in person mattered ('If we're not told, how can we go there?' CS2, AGRI, mother‐in‐law) (SD7). Two substantive efforts to increase self‐help group membership occurred at the launch of interventions and 6 months later. Beyond this, the extent to which Community Service Providers continually tried to find newly pregnant women or to personally invite women to meetings depended on their motivation. Women's own interests were also key to participation. Some had no interest in videos because they could not take up the recommended agricultural practices ('We can't do those things in the house in spite of watching. What is the benefit?' [Self‐help group discussion 1, AGRI]) (SD5). Other women enjoyed learning new things, whether or not they could use this knowledge ('Obviously it feels good to learn something' [CS4, AGRI, pregnant woman]). Women missed meetings due to distance, housework, not having childcare, if they went to their maternal home during the perinatal period, felt uncomfortable sitting in meetings while heavily pregnant or with children, during busy agricultural seasons or bad weather, or if in‐laws made leaving the house challenging (SD8).

#### 3.2.2 Home visits

The trial endline survey (November 2019 to January 2020) found that, across all intervention arms, less than 20% of all mothers of children under 2 years who had seen a video in the 6 months before the survey had also received a home visit from a Community Service Provider. On the other hand, quantitative monitoring data routinely collected during the trial suggested that intervention teams delivered over 98% of planned home visits across all arms (Supporting Information: Table 2). The discrepancy between self‐reported coverage of home visits and monitoring data is partly because the denominators are different: coverage estimates related to all mothers of children under 2 years, whereas monitoring estimates related to pregnant women or mothers of children under 2 who attended meetings. The remaining discrepancy could be due to the fact that there was a 3‐months gap between the last home visits and endline survey, which could have exacerbated recall bias. Qualitative interview data suggest that women's experiences of home visits varied (SD6). Some women did not recall receiving any visits. Others said that Community Service Providers visited mainly to ask them to repeat messages from videos. Other women said Community Service Providers showed videos during home visits and also engaged in active problem‐solving with family members. This variation in women's recollections are likely to reflect genuine differences in visits.

### 3.3 Enablers

In UPAVAN's theory of change, participatory videos and women's group meetings were conceptualised as giving group members relevant knowledge, enabling peer support and, especially in the AGRI‐NUT‐PLA arm, engaging in collective learning and problem‐solving and action with support from the wider community. We found that these objectives were largely met.

Community Service Providers in the AGRI and AGRI‐NUT arms described the main features of their interventions as repeated, dialogue‐based engagement with families to offer demonstrations and use problem‐solving to overcome barriers to adopting new practices (e.g., asking women to ‘try’ new foods or agricultural practices), not just sharing messages through videos (SD9). Community Service Providers' motivation to invite pregnant women to meetings and conduct home visits was affected by the size of their catchment area: although care went into ensuring equitable workloads, some Community Service Providers had to travel long distances to visit remote hamlets. Households in tribal villages were often scattered, making it more challenging to bring women together. Women also tended to be busy and harder to meet at home during the agricultural season. On the other hand, community Service Providers' motivation was increased by interactive review meetings which made them feel part of a team and in which they could resolve problems together.

Women said the interventions benefited them because they gained new knowledge and became more confident to ‘speak up’ about new practices at home (SD10).

I: The way you are able to speak now… Were you able to speak in the same way earlier? P: No… I: Then, why are you able to speak now? P: We have seen and learned, so we are able to speak out. I: What have you seen and learned? P: Seeds are kept this way; one should eat this way; one should stay this way… We have learned and are able to speak… (CS11, AGRI‐NUT, mother)

In the AGRI‐NUT‐PLA arm, Community Service Providers asked each woman in the group to speak. According to Community Service Providers, the practice of speaking and listening to others in the group also made some women more confident to speak at home:

As group members, they have to say one or two lines [in meetings]. Suppose I'm quiet today and you ask me to say something. As a result, I'm saying something. I can get some courage from this! Similarly, their fear slowly decreased. Everyone is participating. (Community Service Provider, AGRI‐NUT‐PLA)

Women said they shared the knowledge they gained from meetings with husbands, in‐laws and, more rarely, with friends or other community members (SD11). Some were afraid of looking like ‘know‐it‐alls' in front of others:

If I tell them more about this, then they will mock me and say [sarcastic tone] ‘You think you understand more than us?! We have also seen the videos, so don't explain them to us'. (CS10, AGRI‐NUT, pregnant woman)

Participatory Learning and Action meetings were open to all community members, and monitoring data suggested that they included women who did not belong to self‐help groups, adolescent girls and occasionally men. Meetings also used games and demonstrations to keep participants engaged in active learning, as described by a Community Service Provider:

We had a meeting on the need to decrease pregnant women's workload. We played a musical chair game in which the woman not able to make it into a seat was awarded a brick. Gradually, when the number of bricks increased, it was difficult for them to hold the weight. We explained that as we get tired while holding bricks during a game, we should think about mothers who carry the extra weight of a child and work so much. How difficult and tiresome it must be for them! They carry 10–12 kg and work all day long for us. Hearing this, participants replied that they did not know about this […] Community Service Provider 1: We also explained this to their mothers‐in‐law […]. Now they forbid their daughters‐in‐law to do heavy work. (Community Service Provider group discussion, AGRI‐NUT‐PLA arm)

In Participatory Learning and Action meetings, group members also identified strategies to address their prioritised problems and took collective and individual responsibility for implementation. Each group implemented a set of prioritised strategies and monitored progress in every meeting. For example, group members in one village decided to practise handwashing with soap before feeding children, counsel mothers‐in‐law and other family members not to impose food restrictions on pregnant women and mothers of children under 2, and give diverse foods to children after 6 months and develop homestead food production. At every meeting, the Community Service Provider discussed the implementation of these strategies and tried to resolve challenges. Some groups also took collective action by organising rallies for the wider community. Overall, enablers specific to Participatory Learning and Action included engaging active learning techniques, in‐built mechanisms to enable problem‐solving, and included more community members than self‐help groups alone.

### 3.4 Exploring intervention mechanisms along the six key pathways to impact

**Figure 3:**
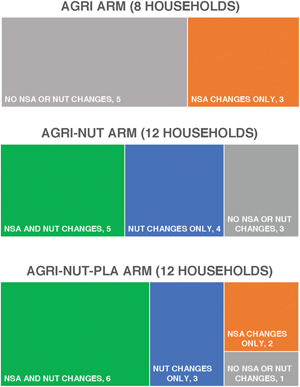
Self‐reported changes in nutrition‐sensitive agriculture and nutrition‐specific practices, by arm, in case studies.

We found that the effects of UPAVAN interventions on agricultural practices were highly heterogenous both between and within‐trial arms. Figure 3 summarises changes in practices seen in the 32 family case studies. In the AGRI arm, three out of eight families who took part in case studies described making changes to agricultural practices. None mentioned changes to dietary practices caused by interventions. Instead, family members commonly said they ate ‘what was available’. In the AGRI‐NUT arm, 9 out of 12 families described changes to dietary practices due to the interventions. Many said that nutrition‐sensitive agriculture would help them save money, be more self‐reliant and eat ‘fresh’, organic food that they would grow and not buy, as opposed to ‘junk’ or pesticide‐laden foods. In the AGRI‐NUT‐PLA arm, 11 out of 12 families described adopting some nutrition‐specific or ‐sensitive practices, or both. The scale of adoption varied greatly. Supporting Information: Figure 2 summarises case studies from six women (two per arm), describing their responses to interventions. We now use these case studies and other process evaluation data to explore how context shaped mechanisms and outcomes along the six ‘pathways’ to improved nutritional status described in the theory of change.

#### 3.4.1 Pathways 1 and 2—Producing food and earning income: It was easier to adopt or improve small‐scale, rainfed homestead garden cultivation for consumption than to production for income generation

In the 32 families who took part in case studies across all arms, 16 made changes to small‐scale vegetable cultivation in homestead gardens, seven made changes to cultivation for income, and nine made no changes to agricultural practices. One Community Service Provider explained that women adopted practices that suited their circumstances:

Suppose there is a farmer. She may not have a courtyard. That's why she may not have done chicken farming. […] Suppose someone wants to do spinach cultivation, but lives in a rented place. She needs some space for that [cultivation]. If she cultivates spinach where she washes [cooking] utensils, then the spinach can't survive because there is lots of water. That's why she may not do it. […] That's why everyone is not successful. But from every video and every meeting, at least all mothers have done something. (Community Service Provider, AGRI‐NUT‐PLA arm)

A minority of women whose families had land and were supportive could influence cultivation in medium to large areas, as described in this case study from AGRI‐NUT (SD12):

Savi explained row planting for paddy to her husband. He adopted it, along with corn and tomato cultivation, and the use of pot manure. The family fed their children corn and sold some too. They cultivated tomatoes by planting seeds at the same time as paddy in June, but could not provide water for more than 20‐30 tomato plants as they had to carry it. Savi's husband recalled: 'When I made the seedbed, she [Savi] came and said, “no, we'll make this kind of seedbed”. We planted in rows. After seven days, we applied fertilizer, manure, everything, and we got good fruits. […] Our family members said, ‘why would we eat vegetables from the market? If we grow vegetables ourselves, then we can eat [them] happily [and] earn money through sales.’

Unlike Savi, however, most women took up cultivation only in their homestead garden. Homestead food production was also a major focus of videos. Homestead gardens were widely regarded as female domains (‘The garden is her responsibility. I'm a man.’ [CS15, AGRI‐NUT, husband]). Women highlighted lack of space, poor irrigation, growing to eat rather than sell, and difficulties in challenging prior practices established by their in‐laws as reasons for not cultivating new crops outside the rainy season or on a larger scale (see, e.g., Rinki and Priti, case studies 1 and 20 in Supporting Information: Figure 2, and SD13). Many nutrition‐sensitive agriculture videos emphasised spacing between plants. Some women felt their land was too small for row plantation and lacked support from their families, so cultivated in ‘women‐controlled’ homestead gardens but not in larger plots:

P: I feel that we cultivate these vegetables only for consumption, so cultivation can be done in any way. My mother‐in‐law […] says that if we plant *brinjal* [aubergine], we can use it for household consumption in curry. […] She also does the plantation work. […] I: Why don't you follow the process of cultivation shown in the video [row plantation]? P: We cultivate in a small place. To follow that process, a bigger space is required. […] Our land is limited. Wherever we get space, we start cultivating spinach or *brinjal*. I: Why don't you do more? P: My father‐in‐law says, 'I can't plough the field. If you want to do it, then go ahead [ploughing is often viewed as a man's activity]'. We remain silent. My husband says, 'Don't do anything, stay at home'. (CS10, AGRI‐NUT, pregnant woman)

#### 3.4.2 Pathway 3—Reducing work: Changes to heavy work in pregnancy do not mean women's work has gone away

Women in all three intervention arms had slightly lower odds of working less than 10.5 h in the last 24 h compared with women in the control arm, but with wide confidence intervals (Kadiyala et al., 2021). What could explain this? Many women in the case studies and self‐help group discussions remembered discussions about the need for pregnant women to avoid heavy work. Several husbands and in‐laws also described changes in social norms about work during pregnancy. These seemed to be driven by a combination of UPAVAN interventions and ongoing government initiatives (SD14):

I: During your pregnancy, was there anyone who said what type of work you should do and what not do? P: Yes, the Accredited Social Health Activist said it, and in the video [we] learned about not lifting heavy things and sleeping under bed nets. I: Who said this to you? P: The Accredited Social Health Activist, mom and our mother‐in‐law … everyone. […] My sister‐in‐law said it. My husband said it. […] I: What did they tell you about housework? P: They say they will do the work. (CS22, AGRI‐NUT‐PLA, pregnant woman)

Still, a gendered division of labour that made daughters‐in‐law responsible for most housework prevailed (SD15), as described by a pregnant participant's father‐in‐law:

I: And who is helping her in housework? […] P: Her husband, I and our children are helping her […] fetching water and cooking. Daughters are also helping her with other work. I: Hm. In what other ways are you helping her? P: What else? That's the work. What should I do? These girls are doing [the work]. How can I? I: How does your son help her? P: What should he do? He is going to work. He is bringing food for her. I: Are you doing something so the child will be born healthy? P: How can I? Girls will know what should be done. (CS17, AGRI‐NUT, father‐in‐law)

Pregnant women whose husbands worked outside the village faced greater difficulties in getting help with domestic and agricultural work, either because they lacked family support or because men working outside the village would say they were unable to help, as described by this couple in a village in the AGRI arm:

I: Are you doing lighter tasks or any heavier jobs? P [Pregnant woman]: I do heavier tasks too. I: You do? They [the Community Service Provider and Accredited Social Health Activist] have told you not to. P: I know, but I still do them. When there's no‐one, I have to do it. I [talking to the pregnant woman's husband]: At times when you don't have work, do you help her? P [husband]: What can I help with? […] I go there [outside the village], so when do I get time to help? (CS4, pregnant woman and husband)

#### 3.4.3 Pathway 4—Making decisions: When women ‘spoke up’, not everyone listened

The UPAVAN trial's endline results showed no consistent effect on women's decision‐making for agriculture or health. Most families valued intrahousehold cooperation (SD16), but women's ability to voice opinions related to farming and have them taken seriously varied considerably between families:

P1: We discuss [cultivation] with everyone in the family and then do it. I: Who does this include? P1: The head of the family, our father‐in‐law [husband, in‐laws and others]. We ask them how to do it and what should we do. We tell them that we have watched videos and that cultivation has to be done in a certain way. They tell us ‘as you have seen the videos, you have to instruct us, and we will do it’. […] I: But who in your family then decides what to cultivate? P2: The head of the family decides. P3: It is not the case in everyone's family. Every family is not the same. Suppose there are people who don't have any father‐in‐law or mother‐in‐law. They do whatever they please. (Self‐help group discussion 6, AGRI‐NUT)

‘Speaking up’ about new agricultural practices did not guarantee that women would be heard. Individual family members' circumstances and personalities mattered. In 16 of our 32 case studies, one or more male household members were involved in daily wage labour or other work outside the village (e.g., Sonamuni's husband, from AGRI, case study 5 in Supporting Information: Figure 2). Husbands who worked outside the village were not always available or interested in supporting cultivation. Even if they were available and ‘listened’ to what women said, they did not always act upon it:

P: She tells [me] but…I don't consider her suggestions and decide as it pleases me. I: Why don't you listen? P: Because… I do what I can. They say ‘we will do that, and that’… but I do what I can. (CS17, AGRI‐NUT, husband)

On the other hand, women with supportive relatives (e.g., Muni, from AGRI‐NUT‐PLA, case study 24 in Supporting Information: Figure 2) were better able to use their new knowledge and confidence to ‘speak up’. A father‐in‐law (AGRI‐ARM) whose wife had passed away described how he supported his daughter‐in‐law's cultivation efforts:

I: Does your daughter‐in‐law give her opinion about what to cultivate? P: Yes, she does. She also does hard work. I: Who decides what to sell? P: My daughter‐in‐law gives her opinions, as does my son. I: Who takes the decision about how money will be spent? P: The decision is also taken by my daughter‐in‐law… and I also take the decision. […] She [daughter‐in‐law] has grown onion and garlic some days ago. Now the green leaves seed will be sown, the land will be cultivated, and after making a seed‐bed, *leutia* (Amaranth leaves) seeds will be sown. […] She waters the plants and I make the seed‐bed. (CS3, AGRI, father‐in‐law)

Gender‐ and age‐related social norms greatly shaped agricultural decision‐making. In most cases, in‐laws or husbands owned the agricultural land, so decisions about cultivation could seldom be made without their involvement. The fact that most families planned crop cultivation and sales ‘by consensus’ also effectively meant that in‐laws' assent was required for all major decisions. Heterogeneity existed even in the context of strong social norms. Some women, like Muni (case study 24, Supporting Information: Figure 2), were able to participate in agricultural decisions and felt listened to by their families. Others were completely excluded from agricultural decision‐making, even though in‐laws expressed great care for them, as described in interviews with a pregnant woman and her mother‐in‐law (CS10, AGRI‐NUT):

Pregnant woman:

P: My father‐in‐law and mother‐in‐law do the plantation work whenever they get an opportunity. I don't do that work. So I don't say [anything about agricultural work]. My mother‐in‐law makes suggestions about cultivation. She says that if we plant *brinjal* brought from outside, we can use it for household consumption, in curry. Otherwise, there is no need to spend money on purchasing *brinjal*. She says this and also does the plantation work. I: Does she ask you about this? P: No. I: How do you feel when she does this without asking you? P: At times she shouts at me and says: ‘only stay at home. don't do anything […]’. So I remain silent. Though she scolds me, she does the plantation work by herself. […]

Mother‐in‐law:

See today I behave badly with her [the daughter‐in‐law]. But I will not stay young forever. A day will come when I will depend on her. At that time, will she look after me? For me, she is both my daughter and son. My daughter‐in‐law is like my daughter and my son. I love her so much.

In sum, women's ability to take agricultural decisions varied considerably both prior to and after UPAVAN interventions.

#### 3.4.4 Pathways 5 and 6—Adequate maternal and child diets: UPAVAN accelerated changes in women's and children's diets

The trial's endline survey found that women in the AGRI, AGRI‐NUT and AGRI‐NUT‐PLA arms had a 21%, 16% and 30% relative increase in the odds of meeting minimum dietary diversity compared to women in the control arm. The trial also found positive effects of AGRI‐NUT and AGRI‐NUT‐PLA on child minimum dietary diversity (19% and 27% relative increase in the odds of children meeting minimum dietary diversity, respectively). The qualitative data provide support for these improvements in women and children's diets. Although some women still experienced dietary restrictions in pregnancy (SD18), practices were shifting through a combination of government frontline worker efforts and the influence of UPAVAN videos and meetings (SD19).

I: The pregnant women in your village and the breastfeeding women in your village, has there been any change in their food habits? P1: Yes. There have been some changes. I: What changes have you seen? P1: Now they are eating [more diverse foods]. They are watching what they should eat in the videos. The pregnant women eat *chathua* [dry grains mix given by the *Anganwadi*] and they also eat fish, meat, and eggs. They also eat green vegetables and spinach… (Self‐help group discussion 6, AGRI‐NUT)

Similarly, many women said that videos had given them knowledge, motivation and confidence to improve child feeding practices (SD20). For example, several described preparing more diverse complementary foods (e.g. Shital, in AGRI‐NUT‐PLA, case study 31 in Supporting Information: Figure 2). Group members mentioned that handwashing before feeding children was also more common because of videos, meetings, frontline workers' initiatives, and ongoing government campaigns (SD21). The Community Service Providers' work was helped by the fact that UPAVAN had purposefully trained government frontline workers to offer similar information to the videos and meetings, and that frontline workers often attended video dissemination and PLA meetings (SD22):

P: Earlier I used to make breakfast for my child, but I used to skip it. […]. In the evening I cooked snacks for the child but I was not eating that. The Accredited Social Health Activists told me to prepare more snacks in the morning and evening so that both I and my child can eat properly. So I followed that. The same thing was shown in the video. (CS10, AGRI‐NUT, pregnant woman)

## 4 DISCUSSION

Our process evaluation found that UPAVAN interventions provided a ‘menu’ of seasonally appropriate crops and strategies to women, which enabled many families to take up nutrition‐sensitive agriculture practices that suited their circumstances. However, uptake of nutrition‐sensitive agriculture was heterogeneous and most often led to rainfed homestead garden cultivation for consumption (pathway 1) rather than cultivation for income and on larger land plots. Small landholdings, lack of irrigation and lack of family support remained key structural and social barriers to cultivation for income (pathway 2). Although norms around heavy work in pregnancy were changing with the convergence of UPAVAN interventions and frontline workers' efforts, younger and less supported women still did considerable housework (pathway 3). Their ability to influence or take decisions about cultivation, income and workload was strongly shaped by the nature of their relationships with in‐laws and spouses, and by the extent to which families were already invested in agriculture versus wage or salaried labour (pathway 4). UPAVAN's strongest effects were in accelerating improvements to women's and children's diets against a backdrop of supportive government initiatives (pathways 5 and 6), primarily by providing women with information, motivation and confidence.

This study adds to a growing body of research aiming to understand factors that influence the delivery of nutrition‐specific and ‐sensitive interventions (Menon et al., 2013). Our process evaluation found that UPAVAN interventions were well‐implemented in the context of an efficacy trial. In the UPAVAN impact evaluation, we hypothesised that the lack of detectable effects along four of the six pathways to improved nutritional status may have been partly due to the strong secular improvements in health, diet and nutritional status seen across Odisha during the trial period (Avula et al., 2020; Kadiyala et al., 2021). The lack of effects on women and children's nutritional status may also be rooted in the intervention's design and social context. Not all women could attend self‐help group meetings, leaving out a substantial proportion of intended participants. Community Service Providers invested variable amounts of time and effort in‐home visits. Women faced considerable barriers to influencing and taking up cultivation, especially on a larger scale and for income.

UPAVAN interventions might be improved by making them more family‐centric. This might include: (a) conducting additional research to understand constraints to women's decision‐making power within the family and how these may be overcome; (b) further tailoring advice to families' specific land, water, labour and time constraints; (c) involving husbands and in‐laws more actively. We elaborate on these suggestions below.

The trial found no effects on gender parity in empowerment in agriculture, but some modest effects on women's decision‐making in agriculture and health‐related activities in the AGRI arm. Previous research has found that relationships between women and their mothers‐in‐law are central to the household economy but also highly heterogeneous (Agarwal, 1997; Allendorf, 2007; Kandiyoti, 1988). This was echoed in our study: some daughters‐in‐law were given little support with housework and were largely unable to take decisions about their diets or workload, while others were given substantial help and shaped some or all decisions. A recent evaluation of a participatory nutrition‐sensitive agroecological intervention in Tanzania also found no effect on women's agricultural decision‐making (Santoso et al., 2019, 2021). Its authors argued for focused research to understand which barriers to increasing women's decision‐making power matter most, and whether addressing these might increase the effects of nutrition‐sensitive agriculture interventions. We aim to address this first evidence gap in forthcoming analyses of UPAVAN data. The need for supportive legal frameworks and working conditions for women in agriculture also remain largely unaddressed in nutrition‐sensitive agriculture interventions and policy across India and South Asia; changing these could greatly increase women's decision‐making power and potentially the effects of community‐based nutrition‐sensitive agriculture interventions (Agarwal, 1994; De et al., 2021).

UPAVAN interventions were developed to respond to constraints that women faced, but production constraints linked to land, water and family support remained a challenge. As a result, nutrition‐sensitive agriculture practices were not taken up universally and were often season‐ and scale‐limited. A recent systematic review of implementation and scale up of nutrition‐sensitive agriculture interventions found that failure to achieve effects on nutritional status and pathways to improving it often arises from a lack of flexibility in intervention design (Di Prima et al., 2022). Some programmes found and overcame similar challenges (Nielsen et al., 2018). Future interventions therefore might emphasise the importance of flexibility in tailoring nutrition‐sensitive agriculture advice to families' specific assets and constraints.

Finally, UPAVAN and similar interventions could be improved by involving other family members beyond pregnant women and mothers taking part in women's groups. Working with self‐help groups provided a potential path to scale up, given India's National Rural Livelihoods Mission aims to reach 70–80 million rural poor households through self‐help groups by 2025 (Government of India Ministry of Rural Development, 2014). However, even after improving their inclusiveness, self‐help groups only reached around half of the mothers of children under 2. In‐laws and husbands are key decision‐makers for agriculture and could have been more actively included in the intervention, rather than expecting young women—many of whom had just moved to their new marital homes—to challenge pre‐existing agricultural practices with only group and Community Service Provider support.

Our study had several strengths. It used diverse data sources including a large body of qualitative data. Methodologically, our approach had similarities with the Programme Impact Pathways approach used in the handful of existing process evaluations of agriculture, nutrition, water, sanitation and hygiene interventions (Mbuya et al., 2015; Olney et al., 2013). Our theory of change served as a map to impact pathways, and we prespecified criteria for reporting components as ‘activated’ or not. Our process evaluation also had several limitations. We did not investigate sources of heterogeneity in agricultural production, household consumption, and women's empowerment quantitatively to test some of the hypotheses generated by our qualitative analysis. Finally, repeated probing for changed practices during interviews and group discussions may have induced socially desirable responses, though we sought to mitigate the effects of this by triangulating data within families and across sources.

## 5 CONCLUSION

UPAVAN's interventions were delivered with high fidelity. They improved women's and children's diets by providing women with information, motivation and confidence. UPAVAN also enabled many families to take up nutrition‐sensitive agriculture practices, but adoption was heterogeneous. Some families started rainfed homestead garden cultivation for consumption, fewer cultivated for income and many were unable to take up practices due to small landholdings, lack of irrigation or family support. UPAVAN interventions and frontline workers' efforts shifted norms towards reducing work in pregnancy, but younger, less supported women still did considerable work. Support from in‐laws and husbands strongly influenced women's abilities to shape decisions about cultivation, income and workload. We recommend furthe research to understand opportunities for increasing women's decision‐making power within families, additional intervention tailoring to address families' specific land, water, labour and time constraints, and involving in‐laws and husbands in nutrition‐sensitive agriculture interventions.

## AUTHOR CONTRIBUTORS

Audrey Prost, Suneetha Kadiyala, Sneha Krishnan, Satyanarayan Mohanty and Helen Harris‐Fry designed the study. Manoj Parida, Suneetha Kadiyala, Satyanarayan Mohanty and Audrey Prost analysed the qualitative data. Audrey Prost wrote the article with structuring inputs from Suneetha Kadiyala, Satyanarayan Mohanty, Helen Harris‐Fry, Suchitra Rath and Sneha Krishnan. All authors contributed to the interpretation of the findings and commented on versions of the article.

## CONFLICTS OF INTEREST

Suchitra Rath, Nirmala Nair, Naba K. Mishra, Ronali Pradhan, Shibanath Padhan and Satyapriya Sahu were part of the Ekjut, VARRAT and Digital Green teams that supported the design and implementation of interventions. The remaining authors declare that there are no conflicts of interest.

## ETHICS STATEMENT

The trial and its process evaluation were led by the London School of Hygiene and Tropical Medicine in collaboration with Indian civil society organisations VARRAT, Ekjut, and Digital Green, research and development consultancy Development Corner (D‐COR), JSI‐SPRING and University College London. VARRAT, Ekjut and Digital Green led the interventions. D‐COR sought consent for participation in interviews or group discussions, audio recording, and the use of pseudonymised data from all participants in writing or using a thumbprint. Ethics approval was granted from the Research and Ethics Committee of the Government of Odisha's Department of Health and Family Welfare (3 September 2016, Letter No. 141/SHRMU), and the LSHTM Interventions Research Ethics Committee (10 October 2016, Reference No. 11357).

## Supporting information

Supporting information.

## Data Availability

Data are available in the article Supporting Information Material.
