# Supplementary material for: Understanding the effects of nutrition‐sensitive agriculture interventions with participatory videos and women's group meetings on maternal and child nutrition in rural Odisha, India: A mixed‐methods process evaluation
Source: Matern Child Nutr. 2022 Jul 19;18(4):e13398. doi: 10.1111/mcn.13398 (PMC9480959; doi:10.1111/mcn.13398)
Supplement: Supplementary file 1 — Supporting information. [file MCN-18-e13398-s001.docx]

**SUPPLEMENTARY APPENDIX**

**Supplementary Table 1:** Characteristics of participants and changes in practices (32 qualitative case studies)

|  | **AGRI**  **(n=8)** | **AGRI-NUT (n=12)** | **AGRI-NUT-PLA (n=12)** |
| --- | --- | --- | --- |
| Scheduled Tribe  Scheduled Caste  Other Backward Class  General Caste | 2  1  3  2 | 4  1  5  2 | 4  1  4  3 |
| Maternal age, median (min-max) | 24 (18-28) | 25 (18-28) | 25 (19-27) |
| Number of videos recalled, median  (min-max)* | 10 (1-42) | 10 (0-14) | 5 (0-8) |

* Participants often had difficulty recollecting the exact number of PLA meetings they went to, and said ‘none’ or ‘many’

**Supplementary Table 2:** Coverage of interventions in the endline survey and monitoring data, by arm

|  | **CONTROL** | **AGRI** | **AGRI-NUT** | **AGRI-NUT-PLA** |
| --- | --- | --- | --- | --- |
| **ENDLINE SURVEY** |  |  |  |  |
| Mothers of children under two years | 997 | 1100 | 1055 | 1139 |
| Active SHG members, n (%) | 458 (45.9) | 530 (48.2) | 537 (50.9) | 546 (47.9) |
| Exposed to any video or PLA in last 6 months, n (%) | 21 (2.1) | 548 (49.8) | 607 (57.5) | 632 (55.5) |
| Saw at least 1 video in last 6 months, n (%) | 21 (2.1) | 546 (49.6) | 607 (57.5) | 627 (55.5) |
| Went to at least 1 PLA meeting in last 6 months, n (%) | 0 (0) | 40 (3.6) | 10 (1.0) | 213 (18.7) |
| Received at least 1 follow-up visit in last 6 months, n (%) | **0 (0)** | **152 (13.8)** | **151 (14.3)** | **220 (19.3)** |
| **MONITORING DATA** |  |  |  |  |
| Number of home visits planned | 0 | 60061 | 57232 | 33703 |
| Number of home visits achieved, n (%) | 0 | 59482 (99.0) | 57051 (99.7) | 33052 (98.1) |

**Supplementary Table 3:** Supporting qualitative data

| N | **Supporting data**  Abbreviations: CSP: Community Service Provider; I: Interviewer; P: Participant; FGD: Focus Group Discussion; CS: Case Study |
| --- | --- |
| 1 | **Training and review meetings increased CSPs’ knowledge about nutrition-sensitive agriculture and maternal and child nutrition**  “CSP: Once the project has called for a review meeting, then we must be present. If you are not present then you’ll regret not attending the meeting. We can get information by phone, but not all the information. […] We cannot actually know what happened in the meeting […] How to show the video, how to do a home visit... Even if we miss only one meeting, we miss out on many things. When we go to the field, we face problems. I: What problems? CSP: If we haven't understood [things] and go to the field for work and someone asks us something, what will we do? If we don't know ourselves, how will we answer others’ questions, how will we clear their doubts? When we have learned, then we can teach others.” (CSP FGD, AGRI-NUT) |
| 2 | **CSPs said they were motivated by helping spread ‘knowledge and development’, gaining recognition from their work in the community, and seeing positive changes in agriculture and nutrition**  “CSP: VARRAT is showing video to SHG mothers, pregnant mothers and mothers who have given birth. We have taken on the superstitions or wrong notions that existed before through the videos. And the things which the mothers were not eating, which they were not doing because of their mothers-in-law, or the superstitions which they had about not eating this or that because it’ll be harmful for the children… By showing videos, most mothers have become aware and are eating, and also giving their children to eat. And the crops which they were not cultivating or the process in which they were cultivating, now they’re following those processes which have been shown in the videos. The SHGs that had broken down, now those SHG are running by showing them the videos for their development. Previously they were not giving money, the mothers-in-law were prohibiting their daughter-in-law to eat some specific things, but now the mothers from the SHG are making their mothers-in-law understand and the mothers-in-law are letting their daughter-in-law to eat those things.” (CSP FGD, AGRI-NUT)  CSP: “Many people came to us [and said] ‘why have you come, do you think you can change the village?’, as nobody had done anything till now. [We said] ‘We are also your village daughters. We will not harm you. We are helping you. You can see my home is there, you all know me.’ […] Now they were saying ‘Sister, we are wrong to scolding you, you helped us to learn about nutrient-rich foods and to cultivate some’. […] They have changed....” (CSP FGD, AGRI) |
| 3 | **Few CSPs had difficulties finding venues for meetings – they used Anganwadi Centres, verandas, other communal spaces or people’s homes.**  “I: Where did you watch that video, please show me. P1: At the Anganwadi. P2: We saw videos at the Anganwadi, and at home also.  I: Do they show [videos] at home? P: Yes. I: How do they show [videos] at home? In whose home do they show? P1: At our house or anybody else’s house... P3: If we five women get together, then we will watch at someone’s home.” (SHG FGD 11, AGRI-NUT-PLA)  “I: Where do you go to watch the videos? P: Where?! We watch [them] in our village. I: Where do they show you the video in the village? P: Near the mango tree. I: Near the mango tree every time, or anywhere else also? P: Inside the rooms also. […] In the temple, in our house also, then near the mango tree, near the mud walls also. I: So you have watched the videos in these places.! P: They show videos to all of us in our house.” (CS4, AGRI, pregnant woman)  “I: In which places do you go and see the videos? P: If there are no people who come to see the video, then they come to show it in our home, and if there are many people in the club house to see the video, then they show us the video there.” (CS3, AGRI, mother) |
| 4 | **CSPs made SHGs more inclusive of pregnant and breastfeeding women, and created additional groups for new members or distant hamlets**  “I: Tell us why you joined the group. P: Due to my mother in-law I attend the meeting. My name is in the group register, that’s why I come to the meeting.” (SHG FGD 12, AGRI-NUT-PLA)  “The people in that village were mostly farmers. […] At that time [when we started], they said instead of mothers-in-law, the daughters-in-law should watch these videos. We, the mothers-in-law are old fashioned and just give flattened rice [to children] […]. Now change is occurring. Everyone’s lifestyle is getting better than before. They can remember all these things, like what food should be used, and in how much quantity. It isn’t sufficient to just ‘fix’ something, there is some appropriate quantity to give. So to remember all those things, the daughters-in-law should watch these.’ Daughters-in-law whose mothers-in-law were coming frequently, those mothers-in-law stopped coming and sent their daughters-in-law more.” (CSP FGD, AGRI-NUT)  “Mothers-in-law also come here and they learn what should be given to their daughters-in-law.” (SHG FGD 11, AGRI-NUT-PLA) |
| 5 | **Women’s own interest in videos and meetings was key to participation**  “P1: I don’t like to see the video, Didi. I: You don’t like! Why don’t you like it? P1: I don’t like. I: Why don’t you like, what is the problem? P1: There is no problem. Those who wants to see the video, they go and watch. I don’t want to see, so I’m not watching. […] Why should I see the video? We can’t do those things in the house in spite of watching. What is the benefit?! There is no benefit.“ (SHG FGD 1, AGRI)  “P1: They show the same videos…they keep repeating that day by day… I: They are showing the same thing? P1: Yes. They are showing the same things daily. If we are interested to do farming then only we will see otherwise why will we waste our time. P2: the same vegetable farming videos. P1: If they show new things then it will be interesting to watch…P3: Yes. P1: If they show the same thing on repeat…who will watch? I: Why aren’t they doing that way? P1: God knows why they are not doing that way.[…] I: Do pregnant women or lactating mothers come to see this video? […] P1: No, they say ‘if are going to cultivate those veggies then we’ll go, otherwise why should we?’  (SHG FGD 3, AGRI) |
| 6 | **What CSPs did during home visits varied greatly.**  **Using home visits to show videos**:  “CSP1: Before showing videos, we do a meeting. At that time, we do a prayer. After that we write down the attendance, like 1 2 3 4, etc.. Suppose we have 10 members and 1 member is absent. Then I ask ‘which mother is absent?’ Everyone says ‘*that* mother is absent’. So we say next time, we will show video at her house. As I said earlier, when a pregnant mother cannot come we go to their homes to show the videos. I: Who else faced this? and went to homes to show videos? CSPs 1 and 2: We all faced.” (CSP FGD, AGRI)  “I: Does she [CSP] come to your house? P: Her house is very far from this place…It is not possible for her to visit at other times. I: How many times she has visited your house? P: Two or three times... […] I: Is she showing you videos by coming to your house? P: Yes. I: Have you gone to the [PLA] meeting? P: I have never been to the meeting. […]. ” (CS 22, AGRI-NUT-PLA, pregnant woman)  “Suppose the mother who has small baby, she can’t come. So after showing the video, I show her personally by giving her some more time, as she has small baby. I tell her that the video is about this or that concept, so watch just a little bit. The video is of 5 to 10 minutes. ‘Just watch the video little bit’. ‘Then watch and tell me how the video is. As you have a small baby, that’s why you can’t go. So I can come to you personally and make you watch. So when you get better, then you’ll also go there and watch video’. Then they say, ‘yes Didi, it is quite nice. You came near me and showed me video. I couldn’t watch the video, I would remain left out’.” (CSP FGD, AGRI-NUT)  “I: When she [the CSP] comes to your house, does she only talk with you or also with other members of your family? P: With me and the other women members of my home. If the women of our neighbourhood get together in our home, then she comes and tells us about all this. And if there is no other woman member in our home, then whomever she finds in the home, she tells them.” (CS3, AGRI, mother)  “I: Has it ever happened that pregnant women or mothers of small children don’t come to watch the video? Do you have any idea about it? P1: They generally show these videos to them at their home. P2: They show them personally. I: They show the video to the pregnant women and mothers of small children personally? P2: Yes.” (SHG FGD 7, AGRI-NUT)  **Using home visits for knowledge or negotiating practices?**  “CSP1: We went for a home visit. Then we asked [the mother] what she remembered about the video she had seen last time. If she answered correctly, then knew she had remembered all the things properly. If she couldn’t answer properly or couldn’t remember properly, then she’d be reminded once again. We told her ‘you’ll remember it in this way, you’ll say it in this way’. Then she’d remember. Then once again we have to ask whether she has remembered properly or not. If she answered properly, we’d know that she has remembered properly. CSP2: *It’s not about remembering. We have to make her do!* We have to observe whether she is doing or not. What will happen if she’ll do? She’ll remember automatically. What do *we* remember? Suppose if we’ll do this work, then automatically we’ll remember. So we’ll ask ‘didi, are you feeding this thing to your baby or not?’ If she says ‘yes, we’re feeding’ then we’ll know that she has remembered that thing properly by watching the video and is using it in her work. So the home visit occurs to see whether she is implementing in her work or not.” (CSP FGD, AGRI-NUT)  **Using home visits to call women to meetings only**  “I: Ok sister, after the video show is over does [CSP] Didi visit your house? P: Yes, she comes sometimes. I: Why does she come?  P: She calls me to join the video show, so I can know things...I: Does she come after you watch the video show?  P: No...she doesn’t.” (CS1, AGRI arm)  **Not visiting, or rarely**  “I: Do they come to your home to call you, before the video show? P: Yes, before the show they come to me and say ‘a video show will happen, please come to that place and watch it’. I: But they never come to you after video show is over? P: No, they don’t come after the show is over.” (CS10, AGRI-NUT, mother)  “P: She [CSP] came once. I: When did she come to your house? P: Last year. I: Why did she come last year? P: To show us the video. I: Ok, yes, she came to show video. But after showing the video, did she ever come to your house after that? P: No.” (CS18, AGRI-NUT, mother) |
| 7 | **Efforts to call women for meetings in person made a difference**  “I: Ok, where did you see that video? P: At school. I: You watched one at school. P: Yes. I: Why don’t you go there [more often]? P: I don’t know. They didn’t call [me]. I: They didn’t call. P: Yes. I: What else? P: Nothing else.” (CS 10, AGRI-NUT, mother)  “If we’re not told, how can we go there? They call us. And then we go.” (CS2, AGRI, mother-in-law)  “I: You said that some women are watching [videos] and some are not. Why is it so? P1: Those who get left or uncalled…they complain ‘we were not called, so why should we go and watch?’. They complain that they have household chores to do. I: Why are they not coming?  P2: They have work to do that’s why! They are saying ‘I have to cook food, feed my child, and my husband will get angry if I go there’.  (SHG FGD 3, AGRI) |
| 8 | **Women missed meetings due to distance, if they were busy with housework, childcare – especially if they were alone with no help - if in-laws prevented them, if they felt uncomfortable sitting in meetings while heavily pregnant or with children, and if seasonal weather disruptions (e.g. heavy rains) made leaving the house challenging.**  “P1: I saw [a video] once in that sister’s house. I have seen five videos in the Anganwadi. I: Who has seen more than 5 videos? Has anyone seen more than 5 videos? [Author’s note: In 2019, after two years of intervention] P2: No, we haven’t seen. We don’t come to this area. It is in this part of the village that videos are shown. I: All of you said that videos are being shown for two years. Do you find a change in the number of people who came to the meetings two years ago, and those who come now? P3: Those who are alone and have work don’t come to attend the meetings. I am alone and the aubergine plantation is still going on in my backyard. I have left everything to come here.”  (SHG FGD 15, AGRI-NUT-PLA)  “CSP1: When it rains and storm occurs, will the people come?! There will be no electricity. How can you show [videos]?! So for that, we have to make-up by showing videos twice or thrice in the day time. Again, people would have work. They say ‘no, we have work in the land, we won’t go to watch your videos. So take our daughter-in-law’. When we tell to the daughter-in-law, they say they’ll eat when we’ll cook food for them, isn’t it! ‘So when will we go to watch videos?’ CSP2: And in the winter season, at the time of paddy harvest, at that time the people go for cutting paddy. Those who remains left, we show them some videos. But other people can’t come and can’t watch the videos. Sometimes a child is crying, sometimes they remain left in the home.” (CSP FGD, AGRI-NUT)  “I: Are they not going to watch video? P1: No, madam. I: Ok, why do you think they are not going to see videos? P2: They will not feel comfortable among the rush of people. How can they watch videos there freely in the rush? P3: They come with small children, holding them.  P4: What should I tell? They have a small child. They cannot come and leave them behind. Even for vaccination they require someone’s help.” (SHG FGD 1, AGRI)  “CSP1: Didi, when the groups were constructed, a lot of mothers were in the group. But occasionally one of them goes for a job, one of them moves to live outside, as a result they get deprived from watching videos. But as they’re the members of the group, that’s why they remain in the group. We have shown that this is why these members cannot watch the video. Because they’re living outside. Someone is going to her father’s house. Someone is going for a medical consultation. So, such problems also arise. CSP2: Some people are doing jobs outside.  CSP3: At the time of birth, mothers go to their own [natal] home. And when the baby is born, she can’t go anywhere for 21 days. She can’t go anywhere for 1 to 2 months. She cannot watch videos for 1 to 2 months. She sees them in the next session.” (CSP FGD, AGRI-NUT)  “I: When did you watch the last 2 videos? P: Six months ago, I watched that last video. It has been one year since I have watched those videos. I: Why are you not going to watch the videos? P: It is because I don’t stay at home or I have works. I: What all work do you have? P: I cut paddy so I have to be there for my work. So when will I go to watch the video?” (CS10, AGRI-NUT, mother)  “I: Where do you go to see the video? P: At the Anganwadi […] they also show videos in my house as I am unable to go now. They call me but I am unable to go, as nobody is here at home.” (CS20, AGRI-NUT-PLA, mother) |
| 9 | **CSPs described the main participatory ‘lever’ of group interventions as repeated dialogued-based engagement with families to offer counselling, problem-solving and demonstrations (e.g. asking women to ‘try’ new foods or agricultural practices), rather than only sharing messages via videos**  “CSP1: […] the point is meeting people door to door. […] if they will not be attending one meeting then we will go to them and inquire, ‘why didn’t you come? What problems did you face?’ If we will help them in their problems then they will listen to us and will come to meetings and also the video. The main thing is that we must go door to door, campaigning. […] Mostly, we need good contact with their family. We needed a good contact with the 1000 days mothers [sic], their husbands and their in-laws. Without any proper contact no one will come. […] I: So when you have first showed the video. which video have you shown? Everyone: Poi Cultivation. I: what was the people's reaction? What did they say? CSP2: They told us that babies shouldn't eat poi as their health will be affected. Many diseases will occur. They said ‘Why are you showing such videos? You shouldn't show such videos’. Then we said ‘You just give you daughter-in-law poi leaves to eat. If any problem occurs, then inform us’. No such problems occurred. I: Who came to watch the videos? CSP2: Their mothers-in-law saw. After that they gave their daughters-in-law poi. Their fathers-in-law shouted and family disturbance occurred. So we again called their fathers-in-law and showed the videos again. So you don't only show videos to SHG members, but also other people? P: Yes, other brothers or boys also watch.” (CSP FGD, AGRI)  “CSP: There was a pregnant woman near our home. She gave birth to a son. Her mother-in-law still didn’t allow her to eat anything […]. She didn’t give her anything to eat. The baby had a heart problem. He was also very light in weight. Then the baby died. […] Now the mother is expecting another child. Some SHG members said: “See, I have also eaten, my child has also become bigger. I’m having no problem.” ANM didi made [the mother-in-law] understand, the ASHA Didi made her understand. I also made understand: “Whose grandson got harmed? Yours. If that child would be here now […] you would be playing with your grandson. Did you get that happiness? You didn’t. So now, will you allow your daughter-in-law to eat properly, will you take care of her properly, allow her to eat iron tablet, not restrict her, allow her to eat fish and meat, allow her to eat vegetables, spinach…? Then the mother-in-law understood. Now she is allowing her to eat spinach, pumpkin, fish, meat, egg. […] We’ll see what will happen, now that they’re allowing her to eat everything.” (CSP FGD, AGRI-NUT-PLA)  **Counselling example** – “There was a woman called S. Her in-laws were not giving her or her child enough food. Then I went to speak to her mother-in-law […] and said ‘you’re saying that she [the child] is eating all kinds of vegetables’. The child’s MUAC was of green colour, but now it was becoming red in colour. The baby was healthy before, but now she was becoming weak. […] The child had had diarrhoea. She said ‘what we should do, didi, so that the baby’s MUAC will come into green grade?’ I said, give it green vegetables, give it ORS each time after they have diarrhoea. The child could eat ORS and the milk was also given. Give the child as much dal, rice and curry as she can eat. Then the mother went to the medical hall [pharmacy or doctor] and returned back. Then the child became well. I told the mother to give the child a lot of vegetables a lot of times and she slowly became well. Now she [her MUAC] is green.” (CSP FGD, AGRI-NUT)  **Demonstration example**: “They [the family] make *handia* (rice beer), so the mother also used to give two spoons of *handia* to her child. […] When I made *chuda santula* (flattened rice with vegetables) for the first time [in her home], I took all the necessary things with me and made it there. Now, that child’s aunt, who is a group member, is quite smart. She brought everything, flattened rice and all. […] When I went again for the home visit, there was still *handia*, and she has given some to her father-in-law, who was sitting there. She had also given her child *chuda santula*. I asked, haven’t you given *handia* this time? The aunt said, ‘No sister, from the day you told us, we brought flattened rice […]. She [the mother of the child] and her child take one bowl and I take one bowl. She isn’t giving *handia* [to the child] anymore.’ That made me very happy.” (CSP FGD, AGRI-NUT-PLA) |
| 10 | **Women perceived the intervention as being mainly about acquiring new knowledge, and occasionally said this made them more confident to ‘speak up’ at home, especially in relation to agriculture**  “I: How do you feel by learning this? P: It feels good. I: Why do you feel good by learning this? P: Obviously it feels good to learn something. I: So… you get to know about new things. What more did you learn from this? P: Nothing more. I: Did you start farming something new after watching the videos? Something that you had never done before? P: No.” (CS4, AGRI, pregnant woman)  “I: How much interest do you have in seeing videos in the future? P1: I have so much interest in learning something, knowing something new… I: Aunty, tell me, how much interest do you have? P2: We are all farmers, we are farming cabbage, tomato, brinjals, Indian spinach...those are shown in video...we were doing that before...by learning something watching these videos, we hope to learn more… I: And what about the [PLA] meeting...How much interested you are? P3: We are much more interested in the meetings... Uneducated mothers-in-law are not understanding their daughters-in-law and giving them punishment. Everybody should learn and understand that. P3: You can learn everything from the meeting about pregnant women, what they should eat, how comfortable they should be...” (FGD, AGRI-NUT-PLA)  “I: After seeing the video, with whom do you discuss? P: With my husband. I: And with who else? P: With my father-in-law, I also say that we will do these cultivations so that it will be better… My mother-in-law has died, so I only discuss this with my husband and father-in-law. I: What do you discuss with your husband? P: What we will cultivate, or what we will cultivate that will better… I: And with your father-in-law? P: With him, I also discuss which crop we will cultivate … then if he replies ‘yes, cultivate that crop’, then we cultivate that crop. […] I: Okay, did you not discuss these things with your father-in-law earlier? P: Yes, but at that time he was taking everything very lightly. He replied ‘yes, do this, if it gives result then it will be okay, and if not then it will be also okay. I: And after seeing the video? P: Now because after seeing the video I learnt various things on cultivation, I told him about these things. I: Is there any other reason that you told him […] Could you tell him all these earlier? P: No, we were not telling him… After seeing the video, we learnt many things, for example if we cultivate in this way then we will get protein.[…]” (CS3, AGRI, mother)  “I: The video which you go to watch, how do you feel? P: I feel good. I: Why do you feel good? P: We get to learn by watching the videos.  I: What will you learn? P: We will learn by watching. I didn’t watch [videos] from before… So, if they would have asked me then, I would have said what I know [about these things]. I: Why are you telling now? P: Now I am watching the video, so I am telling.” (CS16, AGRI-NUT, mother) |
| 11 | **They most commonly described sharing the knowledge from meetings with husbands and in-laws, and less often with friends or other community members**  “I: After watching the videos about farming, with whom do you talk? P: First we discuss about that video. I: With whom? I: With my husband. I: Do you talk to any other person at home? P: Yes, except my husband, with my mother in law. No one else is in our home. I: So you talk with your mother in law. What do you talk about with your mother in law? P: How to do the farming. To help us in farming. I: What do they say? P: They say yes. I: In addition to family members like your husband and mother-in-law, do you talk to anyone? P: No, I don’t talk with anyone else. I: Did you ever talk after watching the videos, like in your group? P: No, we have never discussed. I: Did you discuss with your neighbourhood or your relatives after watching the videos? P: No. I: Why don’t you discuss with others, instead of just your husband and mother in law. Why? P: We only listen to them. I: Did you ever explain the videos to anyone? P: No. M: Did you tell anyone to go and watch the videos? P: Never told anyone.” (CS4, AGRI, pregnant woman)  “P: I told my family members [about the videos]. I: What did you say? P: Today I went to see a video at the Anganwadi. They showed us how to plant spinach, how to water them and how to harvest them. I: Whom did you tell? P: People In our home. I: Who? P: My husband and my mother in-law.’ (CS8, AGRI, pregnant woman)  “I: Did you talk about that video with somebody after watching? P: Yes. With my husband. I: What did you say? P: Only with husband. I: Please tell me, what was the discussion? P: He said yes, we will do agriculture and chicken farming. I: What else? P: Nothing else. I: Nothing else? Did you talk only with your husband, or with someone else? P: No, I didn’t talk with anybody.” (CS14, AGRI-NUT, mother)  “P: After watching that video I talked with everybody. I: Who did you talk with? I: My father, mother, mother-in-law, brother-in-law and sister-in-law. I: What did you discuss? P: About poultry farming, and that children can eat boiled eggs, and green leaves, aubergines, and about Indian spinach cultivation. I: What did they say after hearing from you? P: They said ‘if you watched these, you can try to cultivate’. We are also cultivating green leafy vegetables. I: What else did they say? P: Nothing else. I: Did you discuss with anybody else except your family members? P: I didn’t discuss with anybody else.” (CS17, AGRI-NUT, mother) |
| 12 | **Families with access to land and water were able to tweak some of their existing practices after seeing the videos**  “I: Sister, you tell us. You have been sitting there quietly. What do you farm or cultivate? P1: Rice. I: Rice cultivation… What else do you do?  P1. Vegetables… I: Which vegetables do you cultivate? P1: Potato, onion, garlic. I: You cultivate all that? Okay. What about you? What do you farm or cultivate? P2: Brinjal, cabbage, Spinach, and leafy vegetables, bitter gourds… I: Okay. Since when are you cultivating all these?  P2. In the winters. I: No… What I meant to ask was, since when have you started doing all these? P3: They have been doing these… even before we were here. P1: There have been some changes though, due to the videos. I: What are the changes? P1: We get to know a difference of what distance should we plant trees. We get to know a lot many other things too. We also follow that while planting the trees. I: The change that you went through, it happened as you watched the videos. So, tell me, what results have you been getting due to these changes?  P1. We are getting more yields due to the changes. They tell us and show us how to plant them. We do it according to that. And we were using the fertilizers that were available in the market, they ask us to make our own manure for that. We prepare them and use them. And we are getting better yields and we are also being able to sell some. We are getting good money out of it and we are eating better." (SHG FGD 6, AGRI-NUT)  “I: Is there anything that you can’t do without watching these videos? P1: People used to farm earlier. These videos just make things better.  P2: We plant in our backyards. These videos make people do more works which wasn’t the case earlier. I: Tell me what you were saying. You were working earlier too. What do you think has changed now? P1: We had no proper idea of when to give the fertilisers. After watching these videos, we know when to plant the seeds, when to give water, and when to give the fertilisers.” FGD MAJ AGRI-NUT |
| 13 | **Women highlighted lack of space, poor irrigation, the fact that they did agriculture mainly ‘for consumption’, and the stasis generated by practices previously established by their in-laws as reasons for not cultivating new crops outside the rainy season or on a larger scale**  “I: What do you do with those rice crops? P: We keep some in our house to eat rice and others we sell in the market to bring other eatables such as oil, salt… I: You sell them and bring oil and salt? P: We are poor people; we don't have money to bring all these so we sell the rice.  I: What else do you do? P: Nothing more… I: You just sell and eat? P: Yes, we just sell and eat. I: How much do sell and what do you eat? P: When we sell, we sell around a quintal or two and what's left we eat. I: So you sell rice a quintal or two? P: Yes. I: Now you are not growing vegetables… As you were growing before, what were you doing then? P: We ate what we grew. We have kept goats and all, if there is a shortage then we have to sell our goats and then earn to live our livelihood.” (SHG FGD 3, AGRI)  “I: You people told that you do farming in one season and don’t do in the other because of the scarcity of water. Is it the only reason or there are other reasons as well? P: Water is the reason. I: What are the other reasons? P: Water is the reason. We don’t get enough water to drink in the summers.” (SHG FGD 15, AGRI-NUT-PLA)  “P: We are doing cultivation now. It’s small cultivation, not very big as there is a water problem. We are using waste water to give water in plants, otherwise there is no more water. If there is sufficient water definitely we can cultivate, if there is a tube-well in every house. There is no water supply so we have no interest in agriculture. We have interest in boring a well, after that we can cultivate.” (CS6 AGRI, mother)  “I: What are the things that you plant? P: We plant Javvarisi and eat them. I: Why don’t you do that always, and why only in the month of June?  A: We don’t have direct water connection. The tunnel that supplied water no more supplies water and it’s difficult to carry water and supply it to the lands.” SHG FGD 7, AGRI-NUT  “I: And you sister, you only watched the farming videos… Did you use them? P1: No. I: Why not? P2: Scarcity of water. I: Any other problem?  P3: Also, we don’t have proper place to do cultivation. P4: At the time of groundnut, we do nut farming. We do not cultivate in the summer season because of lack of water. I: That means after watching the videos you are not doing that ? P4: No.” SHG FGD 16, AGRI-NUT-PLA  “I: Which cultivations do you do apart from this? P1: We mostly do greens. P: Why aren’t you doing it now then? P1: We did it recently, the greens. I: Don’t you cultivate anything else? P1: No nothing else. I: Why is that so? What is the problem? P1: The problem of water supply, we need to bring it from quite a distance. I: There is a problem of water now. What do you do during the rainy season? P2: We cultivate a lot during the rainy season. I: What do you cultivate? P2: ginger, onion, potato, brinjal. All these things...” SHG FGD 12, AGRI-NUT-PLA  “P: We don't have land for that. If we had land, then we would do what the [CSP] sister shows us. […] I: Okay. You people said that you are not doing it as you don’t have land to do it. P. Yes. I: Farming needs land but they also show you videos on other things as well. So, what do you do after watching the videos? P: Nothing. What can we do? I: Do you farm anything in your backyard? P: No, we have a small place.”  SHG FGD 6, AGRI-NUT  **“I:** Have you cultivated Indian spinach? P: Yes, if you want, you can see now the branches are cut...now it will be planted elsewhere. I: Why did you do in that method? P: Less expenditure and more harvesting for consuming fresh vegetables...whatever is produced is fresh. I: Has the video show helped doing this? P: Yes, we can know and do by it ourselves properly. I: Ok, do you sell out of it? P: No, I told you before. we do farming only to consume at home...We have seasonal cultivation, and a year-round problem of water. If a canal will be dug in this line, then some benefit will be here. If we get year-round water facility, then I might do year-round cultivation. [to husband] I: What other difficulties you face to do cultivation? P: The main problem for cultivation is water. I: What else? P: First water, after that, other arrangements will be done. We can apply for agriculture loan. But as there is no water, so we did not apply for loan… I: Have you complained to anybody? P: So many complaints have been given by the villagers. Nobody is listening.” (CS1 mother and husband, AGRI arm)  “I: Well sister-in-law, what about yourself? What did you cultivate? P: Nothing. Due to lack of water I could not do anything. Last year I had done. I: What did you do? P1: I cultivated Indian spinach, pumpkin, chilli… I: As per the video that you saw, or just like the other way? P: After seeing the video show. I: Did you cultivate as it was shown in the video? P: This year there was a shortage of water, so I could not do.” (SHG FGD 1, AGRI)  “I: ”Why don’t you sell?” R: ”Sister, we have a large family, that’s why” “FIL: We don’t have that much land.” (CS13, AGRI-NUT, father-in-law) “Are you thinking of doing farming? P: We have no water. I: Water problem… Then, what other problems? P. If we get supply water, then…” (CS13, AGRI-NUT, sister-in-law)  “I: Where do you cultivate in rainy season? P: In our backyard. P: Where else? I: Nowhere else. I: What do you mean by backyard? Where is it? P: Near our house. Like ivy gourd and bitter gourd. I: When did you do this? P: Last year.” (CS14, AGRI-NUT, mother)  “I: What do you plant in rainy season? P: Paddy and maize. I: What else? P: Nothing else. Vegetables can’t be produced in rainy season. I: What about the winter season? P: Aubergines and tomatoes are planted in that season. We planted tomatoes for two years using the tube well water but now, we don’t. I: In the winter season, you said that tomatoes and aubergines are planted. What else? P: Nothing else. We don’t have water.” CS16, NUT-AGRI, mother-in-law  “I: Have you grown any crops there? P: No. I: Why not? P: Because, it is a summer season, and we need lot of water, but there is no water. I: Ok, any other reason? P: No. Other reasons like I am working in home, I have a baby also…I have no support to take care of the baby…so that’s why I am not doing.” (CS19, NUT-AGRI, mother)  “I: Have you done vegetable cultivation related to the videos you have seen? I: No, I haven’t. I: Why not? P: We haven’t done it because now we cultivate solely to consume. M: Did you use to cultivate way before or after they said to do so? P: We used to do it. I: Okay, haven’t the video changed anything for you? P: No.” (CS26, AGRI-NUT-PLA, sister-in-law) |
| 14 | **Norms about heavy work pregnancy were also changing, even among family members not exposed to interventions**  “P1: Before, those old mothers-in-law told their daughters-in-law to work hard to get less pain while giving birth to their children... They are not saying this anymore. This system was not valid. Now doctors are saying everything to the pregnant women. They say that they should not work more. All those things are explained to those old mothers-in-law.” (SHG FGD 11, AGRI-NUT-PLA)  “[Before the pregnancy, my wife] was doing every work, she was bringing water from the well, wood from the jungle, she was cutting the paddy, she making rice from the paddy. But now the government has explained that the baby in the mother’s womb can’t move properly if they will work too much during pregnancy. For instance, bringing water from the well is work, and if she will do that, then it may create problems in her pregnancy. So I tell her to not to do hard work.” (CS15, AGRI-NUT, husband).  “I [to the group] : Has there been any change in the working life of a pregnant woman? P1: The pregnant women are told not to do any hard work. They should not fetch water from the well. They will not grind or beat masala. P2: But if someone is alone in the family, they have to do it. Who else would do it for her? I: So, those who are alone, do it all? P1: They have to. P2: They don’t fetch water. It is strictly prohibited in the videos to do so. P3: Those who have a sister in law or mother in law, they do it for her. If there’s no one in the family, who would do it for her? I: Okay. You told us that those who are alone, they have to do it all. What about those who have a mother in law and a father in law, how much rest do they get and how much do they have to work? P1: They take rest after having their food. I: Who helps them in their work? P1: Their mother in law. The mother in law and the father in law, they all tell her not to anything tough and she is not allowed to pick anything heavy. […] If she has to fetch water from the well, the mother in law does that. If she has to sweep the house, she has to bend, and there would be pressure on her lower stomach, so they don’t let her do that. P1: Now the boys and girls are all weak. We were very strong. P3: In our times, we used to roam around the forest when we were pregnant and we ate many varieties of berries from the forest and we used to give birth to the children without any problem. Can they do it all now? Now they tell us that they can’t even walk. P4: They ask their husbands to take them on their motorbikes.” (SHG FGD 6, AGRI NUT) |
| 15 | **Despite changes in social norms about heavy work in pregnancy, women still face considerable housework, some of it ‘heavy’**  **“I:** Suppose you are at home; a small baby is in the house. If the baby cries, do you help in feeding the baby? P: If it is required to hold the baby, then I do it; otherwise I don’t help her in household work. I: Are you helping in feeding the baby? P: In this case, I help her. I: What else you help her with? For example, your wife is working and the baby is crying, then do you help her in work? P: No.”  (CS1 husband, AGRI arm)  “I: How do you feel… you are doing work and he [husband] is not even helping you… How do you feel? P: I am bound to do it …. Even if I am not able to but, I do it… I: You might be feeling something… you are doing so much work … you even have a small kid with you... P: I get exhausted after doing work, but still I do it.” […] (CS11, AGRI-NUT, mother) “I: Do you think your wife wants to work after watching the videos? P [husband]: She knows no work.” (CS11, AGRI-NUT, mother and husband)  “I: After delivery of your daughter-in-law which family members help her in daily household work? What works she was doing? P: Work means only cooking etc… she was doing the same work as before…she is a girl, what work can she do? After the delivery, the baby was small…she could not work by holding the small baby. When the baby was young, till 21 days she was not doing any work, only cooking partly after that. Now her work is to cook and serve, so she is doing that. […] Except cooking, she is supposed to clean the house. During cultivation, plantation… I: Does she go for agriculture work? P: Yes, sometimes she goes, but not always. Who else do we have? Who will do this? I am old…” (CS20, AGRI-NUT-PLA, mother-in-law) |
| 16 | **Many families valued cooperation, including for farming**  “I: ”Your daughters- in-law also give opinion. Do their suggestions prevail? P: “Yes.” (CS2, AGRI, mother-in-law)  “I: Were you able to speak up about things before? P: Not much before, but after learning, I am able to do things and speak up. […] I: No, do you say anything on selling the yield like now we should sell or anything? P: Yes. I: For example, do you say anything about selling the lady’s fingers that you grow? P: We cannot eat them all, so we sell them. I: But do you say anything about selling? Do you take the decisions? P: Yes. I: Do you say anything about how to spend money from the sale? P: All the money from farming is kept by my father I law. They run the house. All the household needs all ration, food, marriage ceremony, all other emergency work, they take care of it. […] I: Do they listen to you? P: Yes they listen to me. The money is used by everyone for everyone. Oil, soap these all are taken care of by them. I: Do you speak up things like this from before? P: When I was newly married I was not able to speak up, but now I am able to. I: Now why are you able to speak? I: Now I am a mother and now I help in farming vegetables, paddy and all. So I am able to speak. I: Where did you learn? P: From the CSP I learned all the farming technique. I: As you now speak about things with your family, how you do feel? P: I feel good. I do listen to them and they too listen to me. And if everyone listens to everyone and respects everyone’s views, then only the family will run smoothly.”  (CS28, AGRI-NUT-PLA, mother)  “I: Does your daughter in law say anything in all what you talk and decide? P: No. She doesn't get angry. I: It's not about anger, does she tell anything? P: No. I: Does she say ‘this should be done, that should be done’? P: Yes, she tells us. I listen to her, she listens me. We don't have any complaints on that.” (FGD, AGRI)  “I: Does your wife have any say in what and how to cultivate? Does she express her personal opinions to you? P: I never reject doing anything.  I: Do you respect her opinions? P: Yes, I do. If I do not do that, how will my family run smoothly?” (CS17, AGRI-NUT, husband)  “Actually, the give and take of decisions between a husband and wife is necessary, it helps to maintain the perfect coordination.”  (CS26, AGRI-NUT-PLA, husband) |
| 17 | **Many in-laws expressed love and care for their daughters-in-law while also teasing them for wanting to take up cultivation, not involving them in crop and livestock planning and sales, and limiting their decisions to household purchases**  “P: My father in law and mother in law do the plantation work whenever they get an opportunity. I don’t do that work. So I don’t say. […] My mother in law suggests about cultivation… She says that if we plant brinjal bringing from outside, we can use those for household consumption such as curry. Otherwise, there is no need to spend money on purchasing brinjal. She says this and also does the plantation work as well. I: Does she ask you about this? P: No. I: How do you feel when she does this without asking you? P: At times she shouts at me and says: ‘only stay at home. don't do anything… if you bring and plant brinjal we can consume it in curry." So I remain silent. Though she scolds me, she does the plantation work by herself.” […] [then later, the interviewer speaks to the mother-in-law – P: See today I behave her [the daughter-in-law] badly. But I will not stay young for younger forever. A day comes when I will depend upon her. Then at that time, will she look after me? For me, she is my daughter and son both. My daughter-in-law is like my daughter and my son is mine also. So I love her so much.”  (CS10, AGRI-NUT, pregnant woman and her mother-in-law.)  I: Who all takes the decision about what to cultivate? P: My elder son takes all the decision. I: Does your daughter in law speak up? P: No, she does not speak. I: Why doesn’t she speak? P: What would she say? Her thing is to do the cooking and tell us what food to buy. I: Can’t she put her views forward? P: Yes, on what to bring, and from where to bring it.” (CS23, AGRI-NUT-PLA, father-in-law)  “P1: Yes, we do farming collectively and the farm produce is sold in the market by my father in law. I: Who keeps the profit? P1: Father in law keeps it. I: Father in law keeps it, okay. Who decides about the expenditures? P1: The one who keeps it decides about it. I: Don’t you say anything? P1: No I don’t, as they are the bearer of everything.” SHG FGD 12, AGRI-NUT-PLA  “I: Have you said anything about how to spend the money from the sale of vegetables? P: No, nothing is like that. We keep that money only.  I: Don’t you tell anything that this money comes from this and we will spend it one this? P: No… we keep that money and if any urgent situation will come in which we have to spend money, then we spend that money on that. I: They have kept the money like this. R: Yes, madam.” CS3, AGRI, mother)  “I: Have you ever said anything about how the money earned from selling the chickens should be spent? P: When I need some money or when it is needed for a certain job or something. P: Did you say this before? P: What do you mean by before? I: Did you say about this before or do you speak about it now? P: I didn’t use to say before. I: Regarding the expenditure? P: Just recently. I: Why didn’t you talk about this before? P: Earlier I didn’t use to say much about the expenditure and all. I: So why do you say something about it now? P: I don’t know what else to do if I don’t say now! I: Why do you say something about it now? P: If I don’t say something about it when needed, then from where will we get money when it is required! So sometimes I speak.” (CS4, AGRI, pregnant woman)  “I: No, I haven’t said anything, but in our home everything is controlled by the two brothers, isn’t it! So they, the two brothers discussed everything in between them and do whatever is decided. We, the two sisters-in-law don’t say anything in that.” (CS12, AGRI-NUT, pregnant woman) |
| 18 | **Some women still faced considerable restrictions to their diets in pregnancy from their in-laws**  “I: What all were you eating when pregnant? P: Nothing, my mother-in-law doesn’t allow me to eat anything. She just gives me rice and garlic to eat. She says ‘don’t eat much or the baby’s health will be not good’. […] She used to stop me from eating everything. She never gave me vegetables to eat. She stopped me from eating, and all the other oldies of the colony used to come and see what I was eating, and they talked behind my back, saying ‘modern daughters-in-law are not listening to anything, eating everything, whatever they want, that’s why their babies are weak and are being rushed to hospitals every time. In our time, our children were strong because we used to go by traditions’. I: Did you speak with your husband regarding this? P: Yes, I asked him to go and get vegetables so I could eat, otherwise my child would be weak. I said ‘I will not only eat rice, if your mother gives me only rice, then I will go to my mother’s place!’. I: What did he say? P: He brought vegetables. I ate. I: What about meat, fish and eggs? P: I ate everything secretly, hiding, and at meal times I ate as usual.” (FGD 3, AGRI)  “P1: There are in laws who are good and also a lot who are not, so they [the Anganwadi] used to force the pregnant ladies to eat an egg daily to keep the health of the baby progressing. I went to a meeting some days ago where the daughter-in-law was boiling the egg. The father in law shouted that just because she was carrying a baby in her womb, she was determined to eat eggs. Amid all this, they got into a quarrel.” SHG FGD 12, AGRI-NUT-PLA  “I: What happens if you eat Malabar spinach? P1: We don’t know. It’s just that the mothers-in-law don’t let us. P2: They ask us not to eat Malabar spinach. I: Why? Why should you not eat Malabar spinach? P1: The child would be surrounded by bad substances…They say that if you eat Malabar spinach, you would get diseases related to it. P2: They are all the blind beliefs that the people have here. It is gradually going away by watching the videos.” SHG FGD 6, AGRI-NUT |
| 19 | **Family norms about dietary restrictions in pregnancy where changing**  “I: The pregnant women in your village and the lactating women in your village, has there been any change in their food habits?  P1: Yes. There have been some changes. I: What changes have you seen? P1: Now they are eating. They are watching what they should eat in the videos. The pregnant women eat Chatua and they also eat fish, meat, eggs. They also eat green vegetables and spinach…”  (SHG FGD 6, AGRI-NUT)  “I: Does your wife eat the same food that she ate during pregnancy, now that the baby is here? P: Yes. I: Why have you not changed? P: Why should I change...although in villages so many things are forbidden to eat, for a small baby, all the nutrients are needed, including those available with fish or meat. The vitamin which we get from fish and meat, we will not get it from other food sfor the development of the brain of the baby. I: The same food she is eating what she was eating during her pregnancy? P: Yes. I: You have not changed anything. P: No.”  (CS1, AGRI, husband)  “At the time of pregnancy, we give milk, eggs to our daughter-in-law to keep the baby healthy. And about the food items, we give her a little more than what we eat in our family. If she eats well, then she can give birth to a healthy child.” (CS6, AGRI, mother-in-law)  “I: Now during pregnancy what does she [your wife] eat? R- Now, a routine has been prepared for her. Pulses, potato, meat, fish (in small amount). I: Now, she is eating a little more? P: Yes, she is eating more. I: Why is she eating more? P: Now she is pregnant. The baby will remain healthy as well as well as mother. That is why I am giving.” (CS7, AGRI, husband)  “Pregnant women were not eating earlier, but after watching the videos, they’re eating. What happened before was that the mothers-in-law were taking bath at 11 or 12 and were giving food to the pregnant women at 11 or 12. Again they would give food at 2 or 3, and finally at the night. They were eating 3 times. But now the mothers are having tiffin after doing brush their teeth. Then again, they eat at 9 or 10 in the morning, then again, they’re having lunch at 12 or 1 PM. Then in the afternoon, they are having tiffin and again in the night they eat roti or something. But now they are eating more than twice a day definitely. It is happening in front of my eyes now. On that day a mother told me that she was eating three times. I asked about the schedule. She was 6-7 months pregnant. If the mother wouldn’t eat, then definitely the child will suffer, it’ll not gain weight. But now they’re eating. (CSP FGD, AGRI-NUT)  “P1: The mothers-in-law told us that it is good for the child if you only eat once. If you eat twice, the child would catch a cold. Then the Anganwadi taught us everything. P2: Earlier the mothers-in-law were telling pregnant women to eat once and not twice, they would instruct them to drink only water rice and not the plain water. They used to tell us to eat rice once so you have to eat a bellyful. And they also wanted us to eat less at night. We had all those things in our times… I: Were you not able to speak up to them then? P2: No. We could not speak up to our mother in law or father in law. I: Why were you not able to speak up to them? P2: We were afraid… P1: It was a belief that if we ate more, the child would have more to eat and then they wouldn’t be able to digest it. So they would give us rice in the morning once, and then at night, but less food at night. P2: They would give rice and something to go with it. Something light. No curry was allowed. They wouldn’t even allow us to have spinach or any other vegetables. They would only give us rice and garlic. P3: We couldn’t speak anything in front of our mother-in-law. I: When were you allowed to eat everything? P1: Only when the child would be of 1 year and six months. Till then, we would only eat rice and raw garlic. Now they give everything. Rice, dal, and curry… P2: They are giving that now. In our times, it was not allowed. I: Are they eating everything now? P1: Yes.” SHG FGD 6, AGRI-NUT |
| 20 | **Women gained knowledge and motivation to adopt positive IYCF practices**  “P: I have seen the video. It talked about how to mix spinach, potato, tapioca, boil them together and then to feed the children with rice cakes. After watching the video, I boiled the vegetables together and served it either with rice or chapatis. We had no knowledge of these things earlier, but now that we know, we feed them.” SHG FGD 7, AGRI-NUT  “I: Sister, what did you see in the video? P: They showed us how to feed seven-month baby and how to make *chudda* powder for baby, how to make vegetables .They gave us complete information about that. It was very helpful to my baby. I: Did they come to your home to show video? P: Yes, they go to everyone who has small baby and give complete information about food.” SHG FGD 12, AGRI-NUT-PLA  “I: Ok... What are you giving your son to eat now? P: I am giving *suji, simei*, cake, rice and all to eat. My son is also eating apple, grapes or whatever he [husband] is bringing for him. I: What else are you giving? P: He is eating eggs, fish, meat and all. I: Are you giving any vegetables to eat? P: Yes. He is eating boiled *bhanda*, potato, *daal*, rice and all. I: How many times in a day you are giving him? P: I am giving food 3-4 times in a day. I: What else are you giving him to eat? P: He is drinking milk also. I am not giving him cow’s milk. He is drinking mother’s milk. I: You are giving food to you Son to eat. Is it easy for you or difficult? P: I will give no matter its easy or difficult. I: You are doing so much work, now you are picking *mahula* and also you are feeding your child in between that. So the feeding is easy for you, or difficult? I: The food you are giving in the morning is easy for you? P: We knew it from before and we were doing it and after they told us now we are doing it more. I: What are you doing more? What did they say? P: They said to give good things to children for eating. I: Who told you to do this? P: The CSP said. M: You are doing this because CSP told you? P1: Yes.” (CS17, AGRI-NUT, mother)  “I: Did you see the video about how to give her boiled vegetables to [a child to] eat? P: Yes. I: Ok, have you done these for [daughter’s name]? I: Yes, I have done this. I: What have you done? P: I boiled papaya and added carrots too. I boiled everything and mashed them and mixed them. I: Any problem occurred while doing this? P: No problem at all […] She eats well…so plays happily and does everything.” (CS21, AGRI-NUT-PLA)  “In the PLA meeting I learned how to make children eat more. What quantity to feed them. How to feed the child with *chattua*. How to feed eggs to them.” CS25, AGRI-NUT-PLA |
| 21 | **Handwashing was repeatedly mentioned as a practice promoted by videos, CSPs, frontline workers and other campaigns (including *Swacch Bharat*), and widely adopted**  “P: When they show us something in the video, we get to know things about it and due to that, we become aware of a lot of things. They tell us how to feed the children in the video, and we follow that. They showed us to wash our hands with soap and now we wash our hands with soap and feed our children.[…] A. We learned how to clean our hands before feeding the children. We got to know all that after watching the video. We didn’t know that earlier.” (SHG FGD 6, AGRI-NUT)  “I: You have seen many videos. Tell me what are the things you have done after watching the video? P1: After watching the videos, we feed our children after washing our hands with soap. I had no idea about it earlier but learnt this method by watching the video.[…] People are learning. I: What do you think are they learning? P1: They use toilets and don’t go outside. Many things have changed. Toilets have been constructed. People are trying to keep the environment clean by not throwing anything on the roads. I: What are the other changes that you see? P: In food and washing hands. People wash hands before having food and also after coming from the toilets.” (SHG FGD 7, AGRI-NUT) |
| 22 | **Frontline workers offer the same information about maternal and child nutrition as UPAVAN interventions**  “I: Okay, you said that you are listing the instructions of both ASHA sister and the CSP about feeding your children. Who do you listen to the most? P: I listen to both of them. […] In my daughter’s case, I listen to ASHA sister more. Anganwadi sister and ASHA sister both are telling us about this. I: Whom do you listen to the most? R: Anganwadi. M: Didn’t you know what food should be given to a child?  R: Yes, I knew it. But she explains to me properly about this. M: Who explains you? R: ASHA sister and Anganwadi sister.” (CS3, AGRI, mother)  “I: You said that you are pregnant now ,and had two daughters before. What work did you have to do when they were in your womb? P: I was doing the same work then. I did not go outside. I: What else were you doing at home? P: Cooking, cleaning……all these. I: Before pregnancy, you had to do cattle-shed works wiping out the cow dung, but you are not doing these works nowadays. Why you are not doing? P: ASHA Didi said there will be problems during pregnancy if I do lifting work. (CS8, AGRI, mother)  “I: Did you listen something about the child feeding? […] Who came to your house to inform you? P: ASHA sister came home. I: Who else? P: No other person. When I went to take a bath, ASHA *didi* told me about the vaccination date. AWW sister also tells me. They told me to take my son for vaccination.” (CS18, AGRI-NUT, mother) |

**Supporting figure 1:** intervention and trial timeline

**Supporting Figure 2:** Case studies

**
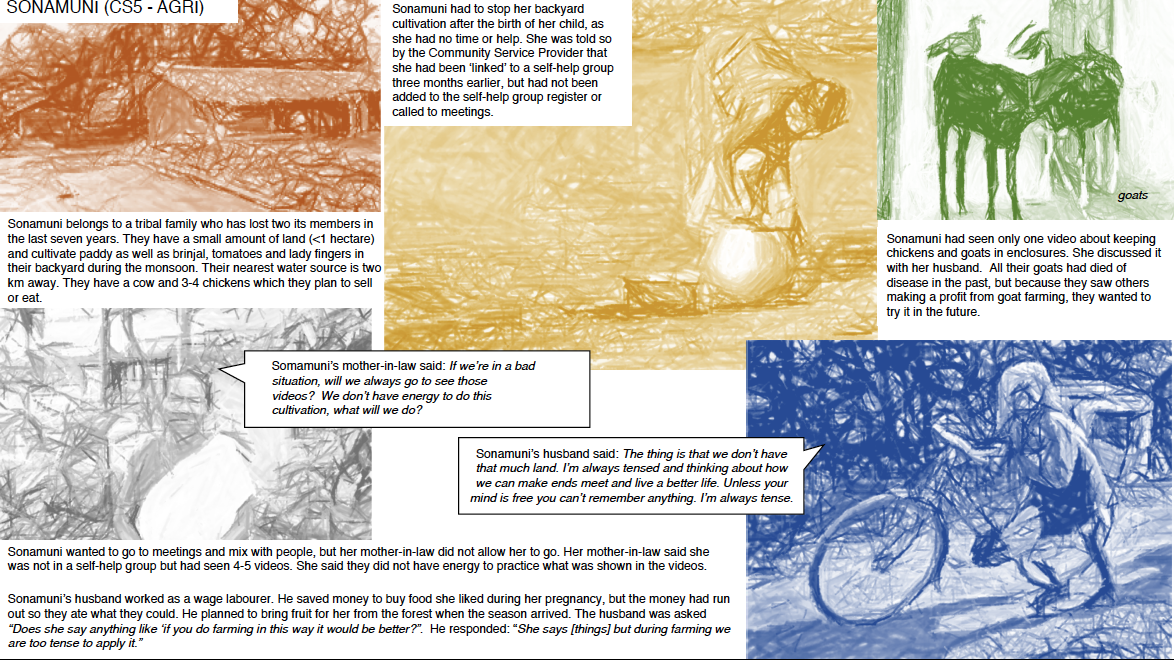
**

**
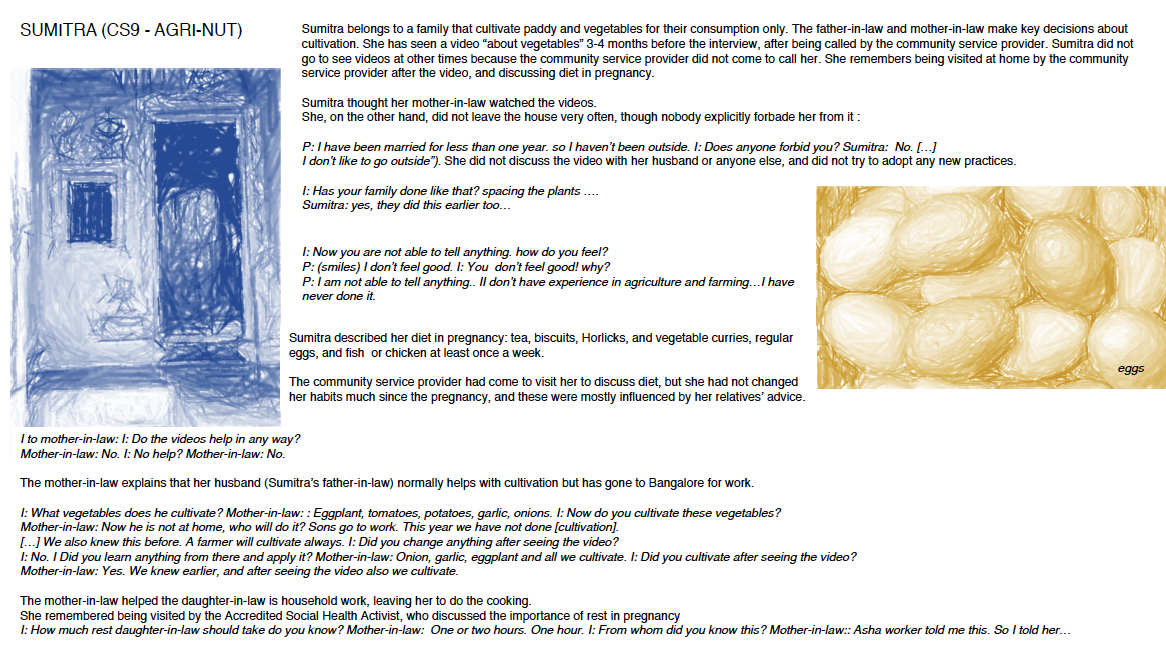
**

**
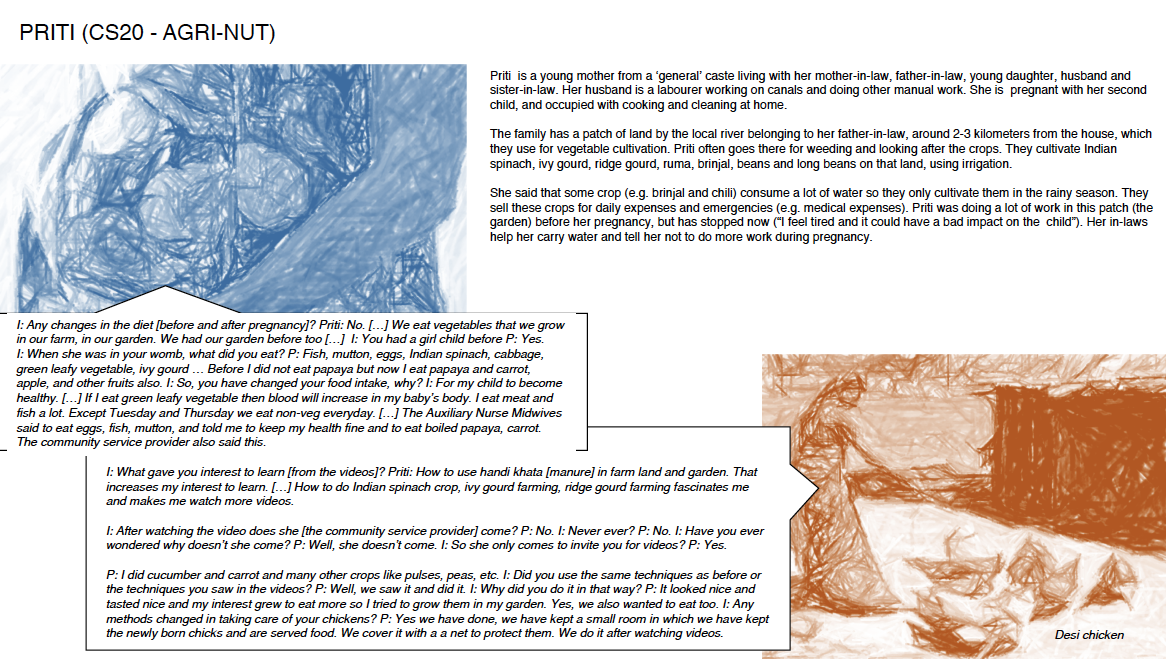
**

**
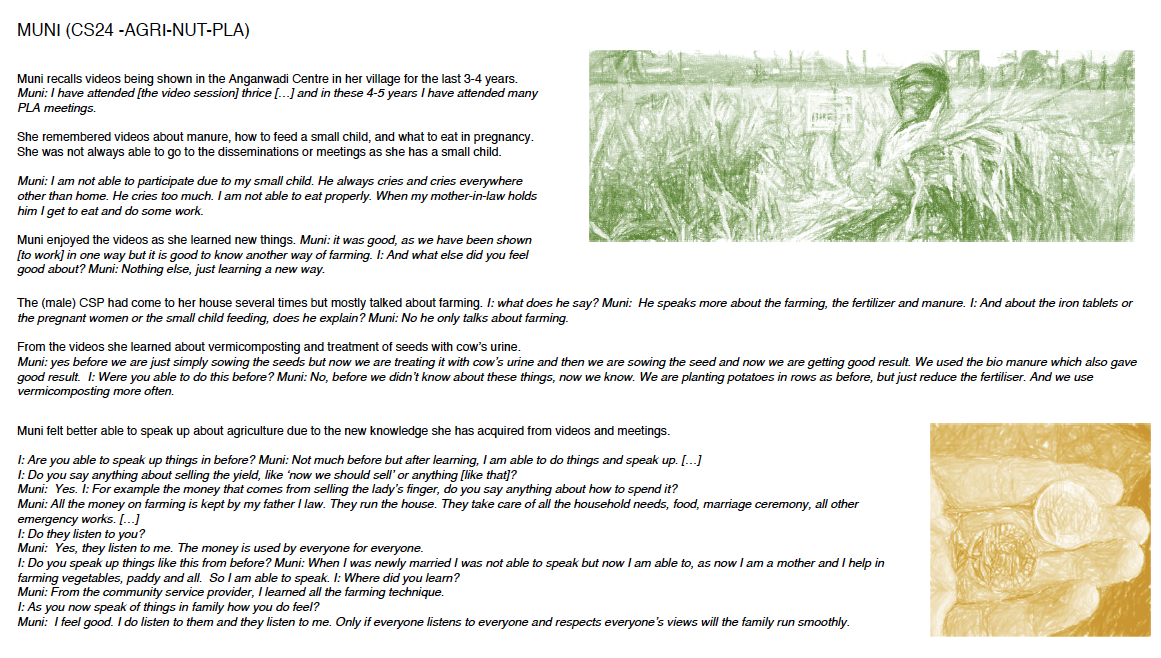
**

**
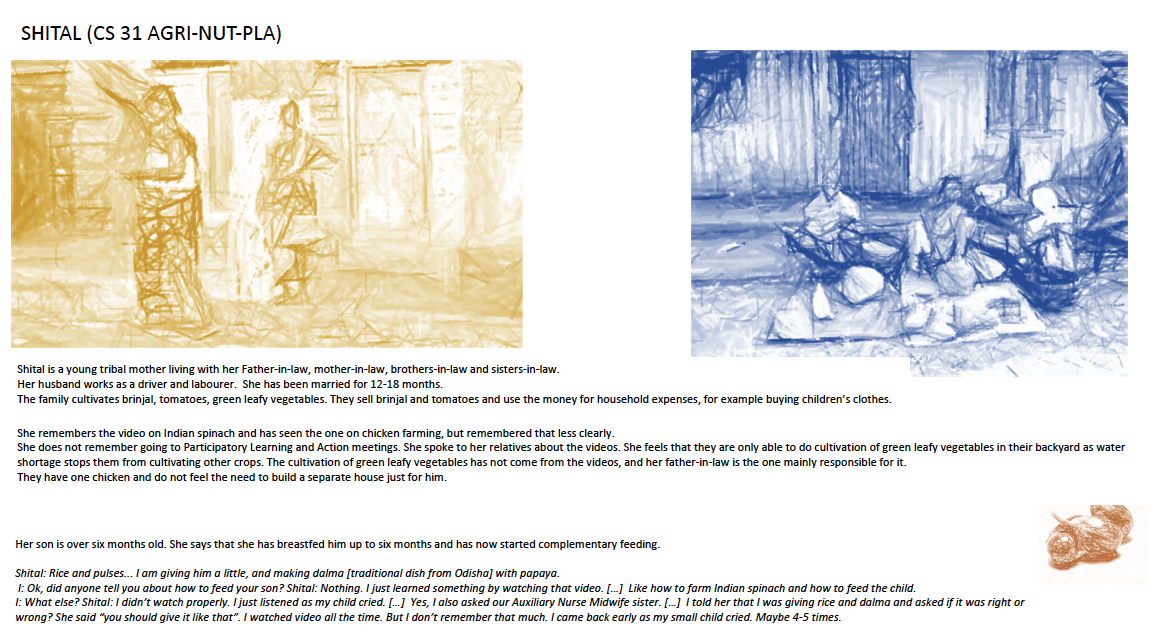
**

**Supporting material:** **Qualitative topic guides**

**TOPIC GUIDE FOR GROUP DISCUSSION WITH SELF-HELP GROUP MEMBERS IN NON-PLA ARMS**

*Welcome and thank you for taking the time to meet with us. We would like to ask you some questions about the video disseminations that have been happening in this village and what you think about them.*

**Attendance in SHG and video disseminations**

- How long have you been meeting as an SHG?
- How long have you been viewing videos as a group, with [CSP’s name]?
  - Who has seen more than five videos?
  - Who has seen more than 10 videos?
- Do you think the number of people coming to see videos has changed since [name of CSP] started showing them? Why?
- Are there any SHG members or other people who do not come to see videos? Who does not come?
  - Women who are not SHG members? Women who live far from the dissemination point? Women who have too much work? Women whose relatives stop them from coming? Women who think the videos are not relevant for them? Any others?
- Are there any pregnant women or mothers of young children who do not come to see videos?
  - Do you think there are many of them?
  - Are they members of SHGs?
  - Why are they not able to come?
- Do other people sometimes attend video disseminations and PLA meetings?
  - Your relatives (in-laws, daughters/sons, others)?
  - ASHAs, AWWs or ANMs?
  - Men?
  - Why do they come to see the videos?

**Effects of video disseminations**

- Can you tell me about any videos you remember and what you remember from them?

*If participants have difficulty remembering, remind them about recently shown videos. Go around the group and ask participants what they remember one by one, then ask who else has seen that video and if they want to add something.*

- Ok, so you said you remembered videos about [say names of videos to remind participants]. What did you do after watching the videos?
  - Did you change anything about what you cultivate, who decides to cultivate what and how?
  - Did you see changes in what others cultivate, who decide to cultivates what and how?
  - How many of you already did these things *before* watching the videos? How many of your started doing these *after* watching the videos?

**Barriers and enablers**

- Which of the things in the videos did you find the easiest to do? Can you tell me a story about this from your or any relatives/neighbour’s experience?
- What do you think helped start these things?
- Did you learn about the practices for the first time through the video?
- Did you get encouragement to adopt the practices from group members or not really?
- Did discuss the practices with other group members after seeing the video?
- Did you discuss the practices with anybody else? Relatives? Neighbours?
- Did you feel more confident to take decisions about the practices because of the video, or not really?
- Did you feel more confident to take decisions about agriculture and diets on your own or together with relatives because of the video, or not really?
- Did you get more support from husband or mother-in-law because of the video, or not?
- Did you get support from other community members, or not?
- What else helped you starting a new practice after seeing the video: availability of land, water, agricultural inputs, money?
- Are there things that you think you would not have been able to do without the help of the videos?
- Are there things you would not have been able to do without the support of group members?
- Which practices did you find hard or impossible to start? Why was this?

**Concluding remarks (for all groups)**

- Is there anything else that you would like to say about how the video disseminations helped or did not help you individually or as a group? *Thank the participants for their time and close the discussion.*

**TOPIC GUIDE FOR GROUP DISCUSSIONS WITH SELF-HELP GROUP MEMBERS IN PLA ARM**

*Welcome and thank you for taking the time to meet with us. We would like to ask you some questions about the PLA and video meetings that have been happening in this village and what you think about them.*

**Attendance in SHG, video disseminations, and PLA meetings**

- How long have you been meeting as an SHG?
- How long have you been coming for PLA meetings with [CSP’s name]?
- How many times have you seen videos?
  - Who has seen less than five videos
  - Who has seen more than five videos?
- Do you think the number of people coming to PLA meetings has changed since [name of CSP] started them? Why?
- Are there people who do not come to PLA meetings? Who does not come?
  - Women who are not SHG members? Women who live far from the meeting place? Women who have too much work? Women whose relatives stop them from coming? Women who think the meetings are not relevant for them? Any others?
- Are there any pregnant women or mothers of young children who do not come to PLA meetings?
  - Do you think there are many of them?
  - Are they members of SHGs?
  - Why are they not able to come?
- Do other people sometimes attend PLA meetings?
  - Your relatives (in-laws, daughters/sons, others)?
  - ASHAs, AWWs or ANMs?
  - Men?
  - Why do they come to see the videos?

**Perceptions of PLA meetings**

- Can you tell me about any PLA meetings you remember and what you remember from them?

*If participants have difficulty remembering, remind them about recently PLA meetings. Go around the group and ask participants what they remember one by one.*

- You said you remembered PLA meetings about [recall previously mentioned meetings]. Can you tell me why you went to these PLA meetings? What did you gain from them?
- What problems faced by women and children did you select (vote for)?
- What strategies did you decide on after this?
- Did having a community meeting help or not really? Why?
- Who has responsibility for the strategies that you decided on?
- Did you face any problems while implementing your strategies? Can you tell us about these?
- What helped you when implementing strategies?
- Were there things that you were able to do as a group that you could not do individually? Can you give an example?

**Barriers and enablers to change after coming to PLA meetings and seeing videos**

- You have also been seeing some videos on agriculture and on the health for women and children. I want to ask you about these.
- Which of the things discussed in the PLA meetings or videos did you find the easiest to do? Can you tell me a story about this from your or any relatives/neighbour’s experience?
- What do you think helped start these things?
  - Did you learn about the practices for the first time through the PLA meetings or videos?
  - Did you get encouragement to adopt the practices from group members or not really?
  - Did discuss the practices with other group members after seeing the video or coming to the meeting?
  - Anybody else? Relatives? Neighbours?
- Did you feel more confident to take decisions about the practices after the PLA meetings and video, or not really? Which do you think helped you the most? Can you tell us more about this?
  - Did you feel more confident to take decisions about agriculture and diets on your own or together with relatives because of the PLA meetings and video, or not really? Which do you think helped you the most? Can you tell us more about this?
  - Did you get support from your husband or other relatives to start new practices after coming to PLA meetings or video, or not really? Can you tell us more about this?
  - Did you get support from other community members to start new practices after coming to PLA meetings or video, or not really? Can you tell us more about this?
- What else helped you starting a new practice after seeing the video: availability of land, water, agricultural inputs, money?
- Are there things that you think you would not have been able to do without the help of the PLA meetings or videos? Which helped most? Can you tell us more about this?
- Are there things you would not have been able to do without the support of group members?
- Which practices did you find hard or impossible to start? Why was this?

**Concluding remarks (for all groups)**

- Is there anything else that you would like to say about how the PLA meetings and videos helped or did not help you individually or as a group?

Thank the participants for their time and close the discussion.

**TOPIC GUIDE FOR FOCUS GROUP DISCUSSIONS WITH CSPs**

*Welcome and thank you for taking the time to meet with us. We would like to ask you some questions about your experiences of conducting PLA and video meetings under UPAVAN project.*

.

1. **Intervention processes and mechanisms**

- Describe your work routine in this project.
- What activities do you undertake? What else?

1. **Personal motivation and experiences**

- What motivated you to this work?
- How did you start this work in your village/cluster? Probes: Whom did you speak to in the village? What decisions were taken?
- What was the community’s reaction when you first started showing videos/conducting PLA meetings?
- Can you tell us about some of the changes you have seen in your village/cluster since you began showing the videos/doing PLA meetings?
- What’s the most enjoyable experience of this work for you?
- What have you learnt from this work?
- What kind of challenges do you face in your work?
  - What else?
  - Anything else?
- Can you describe how you overcame challenges you just mentioned?
- What else helps you in your work?
- Were there any situation that you had to seek support from others?
  - Whom did you ask support from?
  - What did they do?

1. **Video disseminations: Coverage, targets and attendance of 1000-day women**

Now I will ask you some more specific questions about the video disseminations.

- Out of the videos that you have shown so far, what were your two favourite?
- Why did you select these?
- Can you describe how the for the videos were decided on?
- What has been the response of the videos you have shown so far?
- What were the topics the groups really liked and demanded to see more of?
- What aspects of videos did they feel could be improved?
- Please give an example of a video that generated a lot of discussion and debate. What was discussed?
- In a month, on an average how many video disseminations do you conduct?
- How did you decide where to show the videos?
- Roughly can you tell us who participate in these disseminations?
- Which kinds of households do you approach to try and get interested in the Video disseminations / PLA meetings? Why did you choose them?
- Do all attendees participate equally in the discussion during dissemination/meeting? If not, what differences in level of participation do you see between different members, and why do you think so?
- Usually, in a video dissemination how many 1000 day women would attend? How did you encourage them to visit?
- What were the challenges in reaching out to such households?
- How would you encourage 1000 day women to participate in these video disseminations/meetings?
- In your opinion, what are the main barriers to women participating in and attending Video disseminations / PLA meetings?

1. **Home visits**

- Do you visit the homes of the people who have seen the videos?
- What according to you is the purpose of the home visits?
- How many houses do you usually visit after showing the video?
- How did these home visits help?
- How do you engage with the members when you visit the house?
- What would you like to see improved in the work that we do?

1. **Functioning of SHGs**

- On an average, what is the minimum and maximum number of SHGs each CSP has to work with?
- How did you select and support the SHGs?
- Why do you think it is important to work with SHGs for this work?
- What role do SHGs play in facilitating changes at the community level?
- How have other community members supported your work in the village? Can you give an example?
- How have the frontline workers – ASHA, ANM, AWW – engaged with your work in the village?
- What do you think is the biggest advantage of showing videos for improving agriculture and nutrition?
- What is the biggest disadvantage of showing videos for improving agriculture and nutrition?

**5.1 QUESTIONS FOR ARM 3 ON PLA MEETINGS**

You have also been conducting PLA meetings in your villages, now we would like to know more about these meetings.

- Can you describe how was conducting PLA meetings different from your regular video disseminations in the village?
  - Probe: Were there differences in location? Participation of members?
  - Probe: Were different topics discussed?
- What was enjoyable and interesting about the PLA meetings and approach? Please explain.
- Do you think the number of people coming to PLA meetings has increased over the months? Why?
  - Probe: What was the reason behind the increase/decrease in members?
- Are there people who do not come to PLA meetings? Who does not come?
  - Women who are not SHG members? Women who live far from the meeting place? Women who have too much work? Women whose relatives stop them from coming? Women who think the meetings are not relevant for them? Any others?
- Are there any pregnant women or mothers of young children who do not come to PLA meetings?
  - Do you think there are many of them?
  - Are they members of SHGs?
  - Why are they not able to come?
  - What efforts have you taken to encourage them?
- How do we hope to reach out to these 1000-d women through this approach?
  - Do other people sometimes attend PLA meetings?
  - Their relatives (in-laws, daughters/sons, others)?
  - ASHAs, AWWs or ANMs?
  - Men?
  - Why do they come to see the videos?

**Perceptions of PLA meetings**

- What have you found enjoyable and interesting in conducting PLA meetings?
- What helps you in conducting PLA meetings?
- What changes have you seen in group members or others after the PLA meetings?
- What do you think caused these?
  - Information given in the meeting or videos?
  - Group members supporting each other?
  - Relatives of women feeling motivated to adopt new practices because of groups?
  - Self confidence and problem solving skills?
- Can you describe what problems faced by women and children were selected? How did the PLA members went about prioritizing these?
- What strategies did they come up with? How did the groups monitor progress on these activities?
- Can you describe how they went about arranging for community meetings? Did they help or not really? Why?
- What were the problems faced when community members were implementing their strategies? Can you give few examples?
- What helped them to implement these strategies?

**Concluding remarks (for all groups)**

- Is there anything else that you would like to say about how your role in conducting PLA meetings and videos could have helped or did not help make changes in the communities you work in?

Thank the participants for their time and close the discussion.

1. **TOPIC GUIDE FOR PREGNANT WOMEN**

**Introductory discussion**

*Thank the interviewee, ask about her life, her family, their livelihoods, or how her day has been.*

**The intervention and its effects**

- Do you know about any videos and meetings organised by (CSP’s name) in this village?
- Who informed you about these video disseminations? When was this?
- Do you go to see the videos/attend the meetings?
  - *Probe*: How often do you go? Where do you go? If not, why don’t you go?
- How do you feel about seeing the videos/going to the meetings?
- Does the CSP visit you in your home?
  - *Probe*: What do you discuss? Can you give us an example of a discussion? What happened after that?
- Do you talk with others about what you have seen in videos or discussed in the meetings?
  - *Probe*: Can you give me an example of this? What did you discuss after watching videos on agriculture? With whom?
  - *Probe*: Whom did you speak to after watching videos on nutrition?
  - Whom did you speak to after attending PLA meetings? What was their reaction?
- Do you talk to your relatives/friends about what all is shown in the videos/meetings? Did you discuss the practices with other members in your SHG?
- Did you try to convince them to also attend the video dissemination/PLA meetings?
- Did you encourage them to adopt any new practice?

**Nutrition-sensitive agricultural practices**

- Have you seen any videos on different crops or other agricultural practices you can adopt to increase income or for eating in your family? Give some examples
- After watching the videos, in your house, do you grow any crop? If not, why not?
- Can you name any new crops or practices you followed for purposes of consumption? Who decided on these? Who all are involved in this? Are you able to influence the decisions about what to grow for consumption?
- Can you name any new crops or practices you followed for purposes of selling? Who decided on these? Who all are involved in this?
- What do you do with income gained from sale of the products? Who controls the money earned? Were you able to say anything in what to grow or what to do with the income?
- After watching the videos, did you adopt any livestock or change any practices recently? What were they? Why?
- Do you use any livestock products for consumption? Or mainly for sale? What do you do with the income from the sale, if any?
- Whom did you speak to after watching these videos? family? Friends? Anyone else?
- What did you discuss with your family members about growing nutrient-rich crops to either sell or consume?
  - Can you give us an example? Did the video help you?
- Have you changed any crops you grow to eat in the last one year?
  - *Probe*: Who decided on these changes? What did you do? What happened after that?

**Dietary practices and workload of women during pregnancy**

- Have you seen any videos or attend any meetings on dietary practices for pregnant women? Give some examples
- After watching the videos, did you make any changes to the food you ate during your pregnancy? Why?
- Did you change how many times you ate in a day? Did you change how much you ate in a day? Please explain
- Can you tell me about the kinds of food you have been eating during this pregnancy?
  - Probe: any green and orange fruits and vegetables, which, and how often?
  - Probe: fish, eggs, meat, and how often?
- Whom did you speak to after watching these videos? family? Friends? Anyone else?
- What did you discuss? What were their reactions?
- Have you seen any videos or attend any meetings on reducing workload for pregnant women? Why is this important?
- Are you able to take rest during this pregnancy? If so, how? If not, why not?
- What all activities are you involved in? Who supports you with reducing your workload?
- Have you discussed with anyone about how much you work and rest? What was their reaction?
  - Can you give me an example? Did the videos or meetings help?
- Let me share a story with you. Consider Sheela, who is a pregnant women in your neighboring village, she is working in the field and also undertakes household activities. Her family grows rice for mainly consumption and have some poultry, which the father in law sells in the market. What do you think about Sheela?
  - Probe: Whether this is right or wrong? What should she do? Whom should she speak to? Who or what can help her?

**Concluding remarks**

- Can you tell me what kind of changes have you seen in yourself, your family, village or community after watching these videos or attending the PLA meetings? You can also describe about other people’s experiences that you may know of.
- How easy or difficult do you think is for a woman, like you in this village to make such changes in their agricultural work or dietary practices or reducing workload? What makes it easy or difficult?

**End the interview**

- Thank the pregnant woman for her time and thoughts
- Turn the recorder/Dictaphone off
- Remember to make a time to speak to husband and mother-in-law

**TOPIC GUIDE FOR PREGNANT WOMEN**

**Introductory discussion**

*Thank the interviewee, ask about her life, her family, their livelihoods, or how her day has been.*

**The intervention and its effects**

- Do you know about any videos and meetings organised by (CSP’s name) in this village?
- Who informed you about these video disseminations? When was this?
- Do you go to see the videos/attend the meetings?
  - *Probe*: How often do you go? Where do you go? If not, why don’t you go?
- How do you feel about seeing the videos/going to the meetings?
- Does the CSP visit you in your home?
  - *Probe*: What do you discuss? Can you give us an example of a discussion? What happened after that?
- Do you talk with others about what you have seen in videos or discussed in the meetings?
  - *Probe*: Can you give me an example of this? What did you discuss after watching videos on agriculture? With whom?
  - *Probe*: Whom did you speak to after watching videos on nutrition?
  - Whom did you speak to after attending PLA meetings? What was their reaction?
- Do you talk to your relatives/friends about what all is shown in the videos/meetings? Did you discuss the practices with other members in your SHG?
- Did you try to convince them to also attend the video dissemination/PLA meetings?
- Did you encourage them to adopt any new practice?

**Nutrition-sensitive agricultural practices**

- Have you seen any videos on different crops or other agricultural practices you can adopt to increase income or for eating in your family? Give some examples
- After watching the videos, in your house, do you grow any crop? If not, why not?
- Can you name any new crops or practices you followed for purposes of consumption? Who decided on these? Who all are involved in this? Are you able to influence the decisions about what to grow for consumption?
- Can you name any new crops or practices you followed for purposes of selling? Who decided on these? Who all are involved in this?
- What do you do with income gained from sale of the products? Who controls the money earned? Were you able to say anything in what to grow or what to do with the income?
- After watching the videos, did you adopt any livestock or change any practices recently? What were they? Why?
- Do you use any livestock products for consumption? Or mainly for sale? What do you do with the income from the sale, if any?
- Whom did you speak to after watching these videos? family? Friends? Anyone else?
- What did you discuss with your family members about growing nutrient-rich crops to either sell or consume?
  - Can you give us an example? Did the video help you?
- Have you changed any crops you grow to eat in the last one year?
  - *Probe*: Who decided on these changes? What did you do? What happened after that?

**Dietary practices and workload of mothers of children under two years**

- Have you seen any videos or attend any meetings on dietary practices for mothers of children under two? Give some examples
- After watching the videos, did you make any changes to the food you ate during your pregnancy? Why?
- Did you change how many times you ate in a day? Did you change how much you ate in a day? Please explain
- Can you tell me about the kinds of food you have been eating during this pregnancy?
  - Probe: any green and orange fruits and vegetables, which, and how often?
  - Probe: fish, eggs, meat, and how often?
- Whom did you speak to after watching these videos? family? Friends? Anyone else?
- What did you discuss? What were their reactions?
- Have you seen any videos or attend any meetings on reducing workload for mothers of child under two years? Why is this important?
- Are you able to take rest since the birth of your baby? If so, how? If not, why not?
- What all activities are you involved in? Who supports you with reducing your workload?
- Have you discussed with anyone about how much you work and rest? What was their reaction?
  - Can you give me an example? Did the videos or meetings help?

**Dietary practices for children under two years**

- What are you feeding your child (____)?
- How frequently do you breastfeed your child?
- Why is it important for mothers to exclusively breastfeed their children? Were you able to do it?
- Did you have discussions about reducing or sharing workload to enable you to breastfeed?
- Did you see any video or attend a meeting about when to feed your child semi-solid foods? Do you know what you should keep in mind about feeding your child after 6 months?
  - Probe: Do you remember how many times a day a child should be fed and at what age? *Do not probe for right answers, just listen to what the mother remembers.*
  - Is this easy or difficult to do?
  - Are you able to do it for your child? If yes, what helped? If not, what made it difficult?
- What kinds of foods should one feed their children less than two years?
- Let me share a story with you. Consider Sheela, who is a mother of 9 month old boy is working in the field and also undertakes household activities. Her family grows rice for mainly consumption and have some poultry, which the father in law sells in the market. They provide their son with rice and dal twice in a day, and mostly breastmilk.
  - Probe: Whether this is right or wrong? What should she do? Whom should she speak to? Who or what can help her?

**Concluding remarks**

- Can you tell me what kind of changes have you seen in yourself, your family, village or community after watching these videos or attending the PLA meetings? You can also describe about other people’s experiences that you may know of.
- How easy or difficult do you think is for a woman, like you in this village to make such changes in their agricultural work or dietary practices or reducing workload? What makes it easy or difficult?

**End the interview**

- Thank the mother for her time and thoughts
- Turn the recorder/Dictaphone off
- Remember to make a time to speak to husband and mother-in-law

**TOPIC GUIDE FOR HUSBANDS AND MOTHERS-IN-LAW**

**Introductory questions**

Thank the husband/mother in law for giving you time and agreeing to speak with you.

- Do you or anyone from your family attend any videos or meetings where they tell stories and play games in your village? Have you heard about these from someone else?
- What are these videos/meetings about? Since when are these videos/meetings being shown?

**Agriculture, nutrition, and support to pregnant or breastfeeding women**

- Do you or your family engage in agriculture?
  - *Probes:* What types of crops or vegetables and fruits do you grow? What did you do with the produce?
  - *Probes:* Do you consume everything?
  - *Probe:* What proportion of your produce do you sell?
  - *Probe:* What do you do with the income earned from selling?
- Other than farming, and livestock, where else does your family get food from?
  - *Probes:* foraged food, hunting/gathering or fishing? What did you collect and what did you do with forest produce?
  - *Probes:* Purchase from the market such as fish, eggs, milk, fruits.
- Have you changed the foods your family cultivates, collects, purchases, or how these are stored and prepared during (*woman’s name*) pregnancy and after the birth of the baby? What did you change and why?
  - *Probe*: Did you change the foods cultivated? Why?
  - *Probe*: Did you change the foods you collect? Why?
  - *Probe*: Did you change the foods you purchase? Why?
  - Did you change anything about the way you store crops or other foods? Why?
  - Did you change anything about how manure is prepared and handled? Why?
- Have you made any changes to the way in which work is distributed among family members during/ after (woman’s name) pregnancy and the birth of the baby? What have you done?
- Have you made any changes to the way in which your family spends money during/ after (woman’s name)’s pregnancy? What have you done?
- What are some of things you have done to support (woman’s name) so that she and the baby are strong and healthy?
  - Probe: anything related to agriculture?
  - Probe: anything relating to food, grown, collected or purchased?
  - Probe: anything related to workload?
  - Probe: anything related to seeking care for illness?
  - Probe: support with feeding the child (probe further – what kind of support)?

**Perceptions of the intervention**

- Have you heard about the work done by (CSP’s name) in your village?
- What does he/she do?
- Have you participated in any activities with her/him? Which ones?
  - *Probes:* What did you think about the meetings? What was enjoyable and useful about them? What was less enjoyable and useful about them? We are just as interested in negative comments as positive comments, and at times the negative comments are the most helpful.
- Are there any discussions in your family after any member attends these videos and/or meetings?
- Let me share a story with you. Consider Sheela, who is a mother of 9 month old boy is working in the field and also undertakes household activities. Her family grows rice for mainly consumption and have some poultry, which the father in law sells in the market. They provide their son with rice and dal twice in a day, and mostly breastmilk.
  - Probe: Whether this is right or wrong? What should she do? Whom should she speak to? Who or what can help her?

**Concluding remarks**

- Can you tell me what kind of changes have you seen in your wife (daughter-in-law), your family, village or community after watching these videos or attending the PLA meetings? You can also describe about other people’s experiences that you may know of.
- How easy or difficult do you think is for a young woman, like your wife (daughter-in-law), in this village to make such changes in their agricultural work or dietary practices or reducing workload? What makes it easy or difficult?
